# Parafoveal degradation during reading reduces preview costs only when it is not perceptually distinct

**DOI:** 10.1177/1747021820959661

**Published:** 2020-09-28

**Authors:** Martin R Vasilev, Mark Yates, Ethan Prueitt, Timothy J Slattery

**Affiliations:** 1 Department of Psychology, Bournemouth University, Poole, UK; 2 Department of Psychology, University of South Alabama, Mobile, AL, USA

**Keywords:** Reading, eye movements, parafoveal preview, visual degradation, phonology

## Abstract

There is a growing understanding that the parafoveal preview effect during reading may represent a combination of preview benefits and preview costs due to interference from parafoveal masks. It has been suggested that visually degrading the parafoveal masks may reduce their costs, but adult readers were later shown to be highly sensitive to degraded display changes. Four experiments examined how preview benefits and preview costs are influenced by the perception of distinct parafoveal degradation at the target word location. Participants read sentences with four preview types (identity, orthographic, phonological, and letter-mask preview) and two levels of visual degradation (0% vs. 20%). The distinctiveness of the target word degradation was either eliminated by degrading all words in the sentence (Experiments 1a–2a) or remained present, as in previous research (Experiments 1b–2b). Degrading the letter masks resulted in a reduction in preview costs, but only when all words in the sentence were degraded. When degradation at the target word location was perceptually distinct, it induced costs of its own, even for orthographically and phonologically related previews. These results confirm previous reports that traditional parafoveal masks introduce preview costs that overestimate the size of the true benefit. However, they also show that parafoveal degradation has the unintended consequence of introducing additional costs when participants are aware of distinct degradation on the target word. Parafoveal degradation appears to be easily perceived and may temporarily orient attention away from the reading task, thus delaying word processing.

Skilled readers acquire information not only from the currently fixated word but also from the upcoming word in parafoveal vision. When readers can preview the upcoming word, recognition times are faster once this word is fixated compared to when such preview is denied (the so-called *parafoveal preview* effect;^1^ Rayner, 1998, 2009; Schotter et al., 2012). This preview gives readers a head start by allowing them to initiate the processing of the next word before it is directly fixated (Reichle & Reingold, 2013). The preview effect is usually studied with the boundary paradigm (Rayner, 1975), where an invisible boundary is placed before a target word in the sentence. Once participants’ gaze crosses the boundary, the parafoveal preview changes to the actual target word. The preview effect is then calculated by subtracting fixation durations after valid preview from fixation durations after invalid preview when the word is masked (e.g., by a random string of letters).

While the preview effect is highly reliable, there is a growing understanding that it may represent a mixture of preview *benefits* and preview *costs* (Hutzler et al., 2013, 2019; Kliegl et al., 2013; Marx et al., 2015; Vasilev & Angele, 2017). Because boundary experiments typically require an invalid preview condition in which the parafoveal word is masked, such masking may introduce processing costs that overestimate the size of the true benefit. Therefore, the measured effect may consist of benefits due to pre-processing the valid preview and costs due to interference from the parafoveal mask during invalid preview.

Recent research has suggested that degrading the invalid preview mask may reduce its costs (Marx et al., 2015), but adult readers often notice such degraded display changes (Findelsberger et al., 2019; Vasilev et al., 2018). In the present research, we investigated how preview benefits and preview costs are influenced by the awareness of distinct degradation changes occurring at the target word location. In addition, we explored for the first time how degradation may affect parafoveal previews that are phonologically (Experiment 1) or orthographically (Experiments 1–2) related to the target word.

## Preview benefits during reading

Research using the boundary paradigm has explored what type of linguistic information readers obtain parafoveally (see Schotter et al., 2012 for a comprehensive review). For example, it is well-established that English readers acquire useful information from orthographic previews (e.g., Balota et al., 1985; Drieghe et al., 2005; Rayner, 1975). The orthographic preview effect is measured as the difference in fixation durations between a preview that is orthographically similar to the target and a preview that is orthographically dissimilar to the target. Its typical size is around 20 to 50 ms. However, while this effect is interpreted as a benefit from having access to the correct letters in the parafovea, it is also consistent with a cost associated with activating the incorrect letters in the parafovea.

There is also evidence for a phonological preview effect in English (Bélanger et al., 2013; Blythe et al., 2018; Chace et al., 2005; Leinenger, 2019; Pollatsek et al., 1992). However, measuring this effect is complicated by the overlap between orthography and phonology in English. Therefore, the phonological preview effect requires the comparison with an orthographic control, which will inevitably share at least some phonemes with the phonologically similar preview. Because of this, phonological preview effects in English tend to be smaller than orthographic ones and have been estimated to be about 5 ms on average (Vasilev, Yates, & Slattery, 2019). Fitzsimmons and Drieghe (2011) also studied the parafoveal processing of phonology by manipulating the number of syllables of the parafoveal word. They reported that monosyllabic words were skipped more often than disyllabic words, which was interpreted as evidence that readers extract phonological syllabic information early on parafoveally. However, a more recent, higher power replication of this study failed to find the same result, even after taking into account individual differences in reading and spelling ability (Drieghe et al., 2019).

The preview effect is usually assumed to reflect the activation of abstract letter and/or phonological codes (Rayner et al., 2003). For example, using AlTeRnAtInG text, Rayner et al. (1980) demonstrated that preview effects are not based on purely visual information. They found that fixation durations did not differ between trials where the case of all letters changed and trials where the case of no letters changed (see also Slattery, Angele, & Rayner, 2011). They argued that preview effects are due to the processing of abstract letter identities which are case and font independent. However, research has also shown that letter case can have a profound impact on the way that parafoveal information is processed to create preview effects. Slattery, Schotter, et al. (2011) examined preview effects for capitalised abbreviations (e.g., NASA) which were embedded either in normal lowercase sentences or in all-uppercase sentences. They found that the pattern of preview effects depended on whether the abbreviations were visually distinct (i.e., in lowercase sentences) or visually indistinct (i.e., in uppercase sentences). This demonstrates that readers can alter their parafoveal processing based on the awareness of visually distinct areas of text, which has important implications for the incremental boundary paradigm (Marx et al., 2015) and the specific way in which it is experimentally implemented.

## Preview costs during reading

White et al. (2005) were one of the first to directly examine how awareness of display changes may influence the results from boundary studies. They reported that only participants who did not notice display changes showed a foveal load effect (Henderson & Ferreira, 1990). The foveal load effect refers to the finding that when foveal processing difficulty is high (such as when fixating a low-frequency word), readers obtain less information from the parafoveal word due to a decrease in the available attentional resources. In addition, preview effects were larger for participants who noticed the changes. White et al. (2005) argued that the larger preview effects may occur because participants who noticed the display changes “. . . were aware of the difference between the preview and the target [word] (p. 895).” Therefore, display-change awareness may inflate fixation durations due to the detection of perceptually salient changes in the text.

More recently, Veldre and Andrews (2018) found evidence for a foveal load effect only when the invalid preview condition consisted of an orthographically illegal random letter string (Experiment 2), but not when it consisted of an unrelated word (Experiment 1). In addition, the foveal load effect in Experiment 2 was accompanied by a significant parafoveal-on-foveal effect of the random letter string. Importantly, however, the evidence for a foveal load effect in their Experiment 2 was constrained only to participants with higher awareness of display changes. Veldre and Andrews (2018) interpreted these results as indicating that “the interaction between foveal load and preview effects that has traditionally defined the ‘foveal load effect’ may be predominantly due to the preview cost caused by orthographically illegal previews” (p.88).

Slattery, Angele, and Rayner (2011) later investigated readers’ sensitivity to detecting boundary changes using a dual-task signal-detection paradigm. They reported a significant relationship between the sensitivity to detecting a boundary change and the proximity of the pre-boundary-change fixation to the invalid preview. When the fixation immediately prior to the display change was closer to the invalid preview, readers were more likely to indicate that they noticed the change. Furthermore, Angele et al. (2016) used the same paradigm and found that detecting display changes led to inflated fixation durations on the target word. Therefore, these studies suggest that noticing display changes can introduce “awareness” costs that can influence fixation durations.

The possibility that invalid masks may introduce preview costs was first suggested by Kliegl et al. (2013). They re-analysed data from McDonald (2006) and reported that gaze durations (GDs) following invalid letter masks were longer when the previous fixation was located closer to the boundary, as opposed to when it was located further away. Because the visibility of the letter mask increases with greater proximity to the boundary, Kliegl et al. (2013) argued that the longer GDs may be due to interference from processing the random letters in the mask. Interestingly, however, these data mirror to some extent those of Slattery et al. (2011), who found that display-change awareness increased when the pre-target fixation landed closer to the boundary. Therefore, the increase in GD in Kliegl et al.’s (2013) study could have also been related to readers’ awareness of the display changes.

Further evidence that parafoveal masks may cause interference was provided by Hutzler et al. (2013). They analysed fixation-related brain potentials in a task where participants had to read a list of five words and indicate whether the last word (i.e., the target) had previously appeared in the list or not. Hutzler et al. (2013) reported that X-mask previews of the target led to a delay in word processing, which was interpreted as evidence that the mask interfered with foveal word recognition.

More recently, Vasilev and Angele (2017) conducted a meta-analysis of boundary experiments and investigated how the size of preview effects changes as a function of the invalid preview baseline. If the preview effect is not influenced by the choice of baseline, all invalid masks should yield the same effect size. However, if some masks yield larger effects than others, this would indicate that they introduce additional interference costs. Vasilev and Angele (2017) found that the preview benefit increased in size as the invalid preview became less “word-like”—it was smallest for unrelated word previews and largest for X-mask previews. Even so, the difference did not amount to more than several milliseconds in first-pass measures.

The first more direct test of preview costs was done with the *incremental boundary technique* (Findelsberger et al., 2019; Gagl et al., 2014; Hutzler et al., 2019; Marx et al., 2015, 2016, 2017). In this technique, the clarity of the parafoveal preview is gradually reduced by administering increasing levels of visual degradation. If readers obtain benefit from the upcoming word, this benefit will decrease with the reduction in clarity until the valid preview is degraded to such an extent that no further information can be extracted. This method circumvents the use of invalid masks and has been argued to estimate “the absolute size of preview benefits” (Hutzler et al., 2019, p. 23). It is worth noting that the valid preview in such studies is still “masked,” in the sense that it is visually degraded up to the point where no more processing can occur. Therefore, both the classical boundary paradigm and the incremental boundary paradigm involve some type of masking. However, the difference is that the masking does not occur at the level of letter identities (as with invalid masks), but at a lower perceptual level that affects the visual input quality.

**Figure 1.**
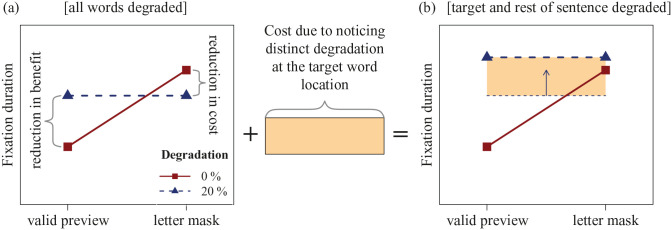
An illustration of the main prediction. When all words are degraded in Experiments 1a–2a (a), the distinctiveness of the target word degradation is removed because no special changes occur there. Therefore, degrading the letter mask should result in shorter fixation durations (reduction in preview cost) and degrading the valid preview should result in longer fixation durations (reduction in preview benefit). However, when degradation begins at the target word location in Experiments 1b–2b (b), there is a cost due to distinct degradation that is specific to the target word. This cost should inflate fixation durations and thus conceal the otherwise expected reduction in preview costs from the mask.

The same degradation method can also be applied to invalid masks to study the preview costs associated with them. When the mask is increasingly degraded, its interference should gradually decrease due to the reduction of its clarity, thus leading to a decrease in preview costs. Once the mask is degraded to such an extent that it can no longer be processed, the cost associated with it should disappear. Therefore, degrading both the valid preview and the invalid mask should eventually converge to the same baseline where the preview is too degraded for any parafoveal processing to occur (see Figure 1a). Hutzler et al.’s (2019) results suggest that this may occur at around 20% of degradation. Therefore, in this paradigm, the “pure” preview benefit is given by the difference between the degraded valid and non-degraded valid condition and the “pure” preview cost is given by the difference between the non-degraded mask and the degraded mask condition.

Marx et al. (2015) were first to show direct evidence for preview costs with the incremental boundary technique. In their study, 4th- and 6th-grade children read sentences in German with three preview conditions (valid, same-shape letter mask, and X-mask). In addition, the target word preview and the remaining sentence were visually degraded in three levels: 0%, 10%, and 20%. Marx et al. (2015) reported that first-pass fixation durations following invalid masks decreased with increasing degradation. Because degradation reduces the clarity of the invalid masks (and thus their potential to cause interference), their results suggest that commonly used masks may overestimate how much benefit readers obtain from the parafoveal word.

Hutzler et al. (2019) subsequently replicated this result in a naming study with adult readers. In their Experiment 2, naming latencies following a letter-mask preview of the target word decreased with increasing degradation, thus replicating the evidence for a reduction in preview costs by Marx et al. (2015). Interestingly, in their Experiment 4, Hutzler et al. (2019) failed to extend the same result to natural reading as GDs on the target word following a letter-mask preview did not decrease with greater degradation. Nevertheless, Hutzler et al. (2019) found this effect on the *pre*-target word, thus demonstrating a reduction in parafoveal-on-foveal effects caused by the letter mask.

Vasilev et al. (2018) also attempted to replicate Marx et al.’s (2015) result in a sample of adult English readers. In Experiment 1, they failed to find conclusive evidence for a reduction in preview costs by administering increasing levels of degradation to letter-mask previews. However, many participants reported awareness of degraded display changes. In Experiment 2, this awareness was explicitly investigated using the display-change detection paradigm (Angele et al., 2016; Slattery, Angele, & Rayner, 2011). The results showed that participants were highly sensitive to degraded display changes, regardless of whether an invalid preview mask or the valid preview of the target word was degraded. The relatively high awareness of degraded display changes was also recently shown in a study with adult readers of German (Findelsberger et al., 2019). In summary, there is mixed evidence that preview costs can be reduced by administering visual degradation. However, as degradation is easily detectible, the mixed evidence could be due to participants’ high sensitivity to distinct degradation changes occurring at the target word location.

## Present research

Previous research has suggested that there are two factors related to parafoveal masking that can influence the results from boundary experiments. On one hand, invalid letter masks may introduce preview costs (i.e., interference) that can overestimate the size of the true benefit (Hutzler et al., 2019; Marx et al., 2015). On the other hand, display changes occurring during invalid previews may also introduce “awareness” costs if readers frequently notice them (Angele et al., 2016; Vasilev et al., 2018; see also White et al., 2005). The existence of preview costs has important implications for reading research, as parafoveal processing plays an integral part in most eye-movement models of reading (e.g., Engbert et al., 2005; Reichle et al., 1998; Snell et al., 2018). Because such models are evaluated against studies that have used invalid masks, it is important to reliably show the existence and likely magnitude of preview costs.

Marx et al.’s (2015) incremental boundary technique is very useful for this purpose as it makes it possible to experimentally reduce the costs associated with invalid masks, thus proving their existence. However, one unintended consequence of this technique is that adult readers are often aware of the parafoveal degradation used in such studies (Findelsberger et al., 2019; Vasilev et al., 2018). In this paradigm, degradation always starts at the target word location and continues all the way to the end of the sentence. However, because the target usually appears somewhere in the middle of the sentence, this creates two different parts of the text: while the first half (before the target) appears normally without any degradation, the second part contains visually distinct degradation that starts at the target word location. Critically, once the target word boundary is crossed, all degradation disappears as the display-change boundary is in the same text location as the distinct degradation boundary. Therefore, if there is a processing cost associated with noticing the distinct degradation and/or boundary-change starting at the target word, this would be reflected in longer fixation durations on the target. We hypothesised that this additional cost (henceforth, “distinct degradation cost”) may confound any attempts to reduce the preview costs of invalid masks and explain some of the mixed results.

The main goal of this research was to test how the distinctiveness of target word degradation influences preview benefits and preview costs. In two experiments, we manipulated what part of the sentence was degraded to make the target word degradation either perceptually distinct or not distinct. In Experiment 1a, we modified the original incremental boundary technique by visually degrading all words in the sentence prior to their fixation. Because the target was just another word in the sentence, this effectively eliminated the distinctiveness of the target word degradation and concealed any special changes happening there. In other words, participants were aware of the presence of degradation across the whole sentence, but did not perceive any special changes at the target word location. In Experiment 1b, we used the original incremental boundary paradigm where target word degradation is perceptually distinct because only the target word and the remaining sentence are degraded. This effectively highlights degraded previews because they occur only in the second part of the sentence. To be clear, participants in both experiments are aware of degradation changes happening at the target word location. However, the critical difference is that in Experiment 1a the target word degradation is not distinct because all words are degraded and thus the presence of degradation is “normal.” Experiment 2a and 2b attempted to replicate the key results from Experiment 1a and 1b, respectively.

We expected to observe a reliable reduction in preview costs only with the modified incremental boundary paradigm where the distinctiveness of target word degradation is eliminated (Experiments 1a and 2a) but not with the original paradigm where target word degradation is perceptually distinct (Experiments 1b–2b). This is because the awareness of distinct target word degradation in Experiments 1b–2b was expected to add additional costs (see Vasilev et al., 2018) that would inflate fixation durations and thus conceal the reduction in preview costs from the degraded mask. This is illustrated in Figure 1. A secondary goal was to investigate how parafoveal degradation and display-change awareness may affect non-word previews that are orthographically or phonologically similar to the target. This is important as previous research has only considered how degradation may affect previews that are either completely valid or completely invalid.

## Experiment 1a

Experiment 1a tested whether the preview costs associated with letter masks can be reduced by visually degrading them when the distinctiveness of target word degradation is eliminated. To do this, we modified the incremental boundary paradigm by degrading all words in the sentence and placing an invisible boundary before each word. Once each boundary was crossed, the degraded preview was permanently replaced by the undegraded word. This eliminated the distinctiveness of the target word degradation/change because all words in the sentence had the same degradation/change. Therefore, there was nothing unusual at the target word location that participants would perceive as different or distinct.

This manipulation bears some resemblance to Warrington et al.’s (2018) Experiment 2, where all words were presented in a lower contrast before their fixation by placing an invisible boundary before each word. However, Warrington et al.’s (2018) experiment focused on how the reduced contrast affects the amount of parafoveal processing that occurs in readers of different age groups. As such, it is not informative of whether a similar manipulation can hide the distinctiveness of target word degradation changes. Therefore, Experiment 1a is the proof of concept of whether such a manipulation would work.

The experiment used a 2 × 4 within-subject design with parafoveal degradation (0% vs. 20%) and preview type (valid, orthographic, phonological, letter mask) as the factors. We predicted an interaction between the preview effect (valid vs. letter-mask preview) and degradation. This was because we expected a decrease in fixation durations when letter masks are degraded (reduction in costs) and increase in fixation durations when valid previews are degraded (reduction in benefit; see Figure 1a). In addition, we expected that degrading the orthographic and phonological previews should reduce the amount of benefit that readers obtain from them similar to the valid preview condition in Figure 1a. This should also result in an interaction between each of the two effects and degradation.

### Method

#### Participants

Sixty-four^2^ Bournemouth University students participated for course credit or a payment of £5 (50 females). Their mean age was 19.8 years (*SD* = 2.1 years; range: 18–32 years). Participants were native speakers of British English who reported normal or corrected-to-normal vision and no prior diagnosis of reading disorders. Participants were naïve as to the purpose of the experiment. Ethical approval for the study was obtained from the Bournemouth University Research Ethics Committee (protocol No. 14059). All participants provided informed written consent.

#### Materials and design

**Figure 2.**
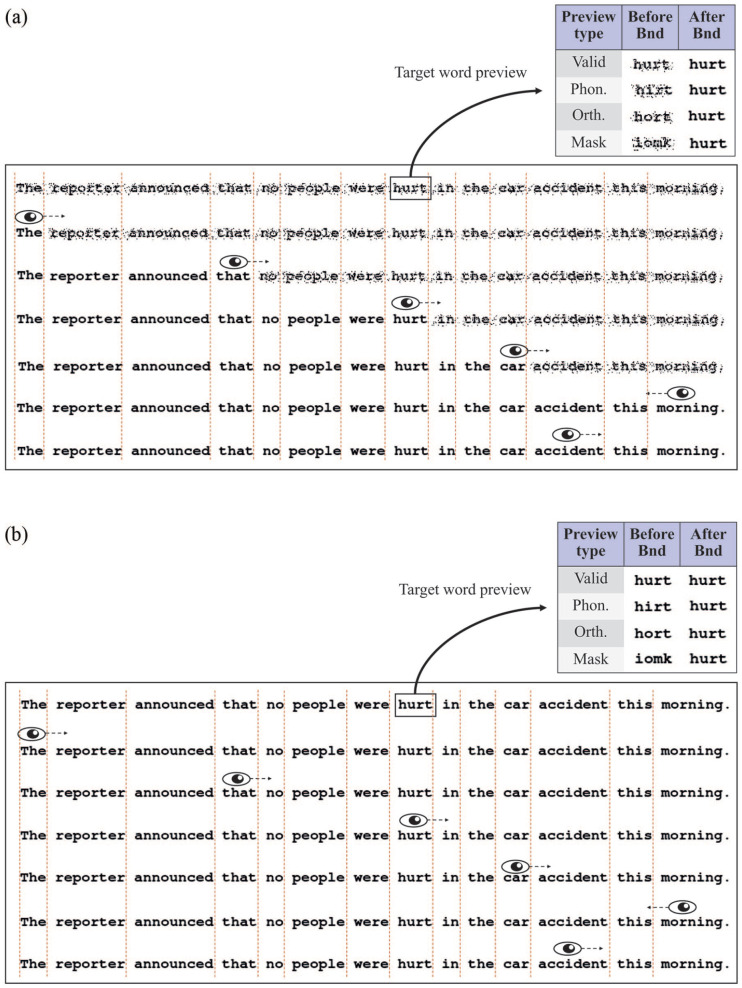
An illustration of the gaze-contingent manipulation in the modified incremental boundary paradigm where all words in the sentence are degraded (Experiments 1a and 2a). In each panel, the rectangle shows the sentence reading condition and the small square shows the parafoveal preview manipulation on the target word (“hurt”). Vertical dotted lines indicate the invisible boundary before each word. In the degraded condition (a), each word was visually degraded before it was fixated. Upon crossing a boundary located before each word in the sentence, the word changed from degraded to non-degraded and remained non-degraded for the rest of the trial. In the non-degraded condition (b), only the parafoveal preview of the target word was manipulated (i.e., this is identical to Rayner’s (1975) classical boundary paradigm). For all other words, no visual change occurred on the screen. Note that the phonological preview condition was removed from Experiment 2a.

The stimuli consisted of 80 English sentences (see Figure 2 for an example and Supplementary Material 2 for all the stimuli). Their length was 14.3 words on average (range: 10–17 words). In each sentence, there was a target word whose parafoveal preview was manipulated using the boundary technique (Rayner, 1975). The target word was 4.78 letters long on average (*SD* = 0.62; range: 4–6 letters). The position of the target word in the sentence was varied, but it was never one of the first or last two words in the sentence (mean position: 7.6 words). The pre-target word was 5.7 letters long on average (*SD* = 1.29 words; range: 4–8 words). All target words were single-syllable words.

There were two within-subject factors that were crossed in the experiment: *target word preview* (valid, phonological, orthographic, letter mask) and *parafoveal degradation* (all words degraded prior to their fixation vs. no words degraded prior to their fixation). The four target word preview conditions are shown in Figure 2. In the valid condition, the preview was the target word itself and no visible change occurred on the screen. In the phonological and orthographic preview conditions, a pseudo-homophone (PH) and pseudo-word (PW) orthographic control were selected for each target word from the ARC non-word database (Rastle et al., 2002). The PH served as the phonological preview of the target word and the PW served as the orthographic preview. Both types of non-words were pronounceable and contained no illegal bigrams. The PHs and PWs were matched exactly to each other and to the target word on number of letters (*M* = 4.77) and number of phonemes (*M* = 3.41). In addition, the PH and PW masks did not differ significantly on the number of orthographic neighbours, PH: *M* = 4.28; PW: *M* = 4.13; *t*(152.7) = 0.31, *p* = .75, or phonological neighbours, PH: *M* = 14.28; PW: *M* = 13.7; *t*(157.7) = 0.44, *p* = .65. Both neighbour types were obtained from the ARC non-word database (Rastle et al., 2002). Finally, in the letter-mask preview condition, a pseudo-randomly generated string of letters was used. Each letter in the mask was matched in terms of ascenders and descenders to the target word. The letter masks were generated in the same way as in Vasilev et al. (2018).

In the parafoveal degradation manipulation, all words in the sentence were either degraded or not degraded prior to their fixation (see Figure 2). In the degraded condition, every word was visually degraded until participants crossed an invisible boundary located just before that word. Once each boundary was crossed, the word permanently changed from degraded to non-degraded. The degraded stimuli were created with the same script used by Marx et al. (2015). In their study, Marx et al. (2015) performed visual degradation by randomly exchanging black and white pixels in the stimuli image. A visual degradation level of 20% was chosen for comparability with previous studies (Marx et al., 2015; Vasilev et al., 2018). Recent research has shown that this level of degradation completely prevents the processing of valid preview information from the parafovea (Hutzler et al., 2019). In the non-degraded condition, all words appeared normally and without any degradation. Note that the non-degraded condition is equivalent to the classical boundary paradigm (Rayner, 1975) because visible display changes occurred only on the target word.

#### Apparatus

Participants’ eye movements were recorded with an EyeLink 1000 eye-tracker at a sampling frequency of 1,000 Hz. The resolution noise was <0.01° and the velocity noise was <0.5° on average. Viewing was binocular, but only the right eye was recorded. A head rest was used to minimise head-movement artefacts in the data. The experiment was programmed in EyeTrack v.0.7.10h (Stracuzzi, 2004) and was run on a PC with a Windows XP operating system.

The sentences were displayed on a 20″ Iiyama Vision Master Pro 510 monitor with a screen resolution of 1,024 × 768 pixels and a refresh rate of 150 Hz. The sentences were formatted in a bold, monospaced font (“Courier”) and appeared as black text over white background. Each sentence appeared on a single line in the middle of the screen. The width of each letter was 11 pixels. The distance between the eye and the monitor was 65 cm. At this distance, each letter subtended approximately 0.36° per visual angle.

#### Procedure

Participants were tested individually in a session that lasted for about 20–30 minutes. Participants were instructed that some of the sentences may appear a bit “fuzzy,” but that they should try to ignore that and read them as normally as possible. The experiment started with a 3-point horizontal calibration. The calibration error was then monitored with a drift check before each trial and was kept at <0.30° throughout the experiment. The calibration procedure was repeated whenever necessary to maintain this level of accuracy.

The experiment started with six practice trials (half of them presented in the degraded preview condition and the remaining half in the non-degraded preview condition). The experimental trials were then presented in a pseudo-random order. Before the start of each trial, a black gaze box was presented at the location of the first letter in the sentence (50 pixels from the left margin of the screen). Once a stable fixation inside the gaze box was detected, the box disappeared, and the sentence was presented on the screen. An invisible boundary (Rayner, 1975) was located at the first pixel of the empty space immediately preceding each word. Once the gaze position of the eye moved to the right of each boundary, the parafoveal preview changed to the actual target word. Display changes were completed on average within 6.22 ms of the eye crossing the invisible boundary (*SD* = 1.05 ms). Forty percent of the sentences were followed by a “Yes/No” comprehension question. For example, in the sentence “The reporter announced that no people were hurt in the car accident this morning.,” the comprehension question was “Were any people injured in the accident? Yes/No.”

#### Data analysis

First fixation duration (FFD), single-fixation duration (SFD), and GD were analysed as dependent variables in the target word analyses. FFD refers to the duration of the first fixation made on a word. SFD refers to the fixation duration when the target word is fixated only once during first-pass reading. GD is the sum of all first-pass fixations on the target word before moving on to another word. A few global reading measures were also analysed to test if the degradation manipulation affected reading behaviour: sentence reading time, mean fixation duration, number of fixations, and saccade length. We also report two post hoc analyses in Supplementary Material 2: (1) parafoveal-on-foveal effects on the pre-target word and (2) target word analyses with additional measures (skipping probability, regression-in probability, and regression-out probability).

Statistical analysis of the data was performed with (Generalised) Linear Mixed Models ([LMMs] using the *lme4* package v.1.1-21 (Bates et al., 2014) in R v.3.51 (R Core Team, 2018). Fixation durations were log-transformed in all models. Random intercepts were added for both participants and items (Baayen et al., 2008). We first tried to add random slopes for both independent variables (Barr et al., 2013), but the models converged only with a Degradation random slope for subjects (the only exception was the saccade length model where the random slope had to be removed). Sum contrast coding was used for the sentence degradation condition (0%: 1; 20%: −1). Custom contrast coding was used for the parafoveal preview condition with the following comparisons: valid versus letter-mask condition (invalid preview effect), orthographic versus letter-mask condition (orthographic preview effect), and phonological versus orthographic condition (phonological preview effect). The results were considered statistically significant at the .05 level if the |*t*| and |*z*| values were ⩾1.96.

### Results

All participants had comprehension accuracy greater than 81.2% (*M* = 93.2%; *SD* = 22.9%), thus indicating that they had no problems understanding the sentences. There were no significant differences in comprehension accuracy (all |*z*|s ⩽ 0.81). After the experiment, participants were shown a mouse simulation of the display changes and asked to indicate whether they saw degraded preview changes and letter changes in the non-degraded trials. We did not distinguish between different types of degraded previews because readers notice only the presence of degradation and not the specific preview (e.g., valid vs. letter mask) that is degraded (Vasilev et al., 2018). In the non-degraded conditions, participants were asked separately about noticing single-letter changes (orthographical and phonological previews) and whole-word letter changes (letter-mask previews). However, many participants were confused about which of the two they saw, so these were pooled together as “letter changes.” All but one participant indicated that they noticed the parafoveal visual degradation. This was expected as participants were explicitly told about the presence of degradation beforehand. In contrast, they were not informed about the presence of letter changes in parafoveal vision. Only 12.5% of participants reported noticing such letter changes in the non-degraded sentence condition.

Fixations shorter than 80 ms that occurred within one letter of another fixation were combined with that fixation. All other fixations shorter than 80 ms were discarded. Trials with track losses or blinks on the pre-target or target word were excluded from further analysis (12.5% of the data). In addition, trials in which the target word boundary was not crossed in a forward saccade or the display change was completed after fixation onset of the target word were also excluded from the data (13.5%). Trials with fixation durations longer than 800 ms for FFD and SFD or longer than 1,600 ms for GD were discarded as outliers from all analyses (0.14%). This left 73.8% of the data for analysis.

#### Target word

**Figure 3.**
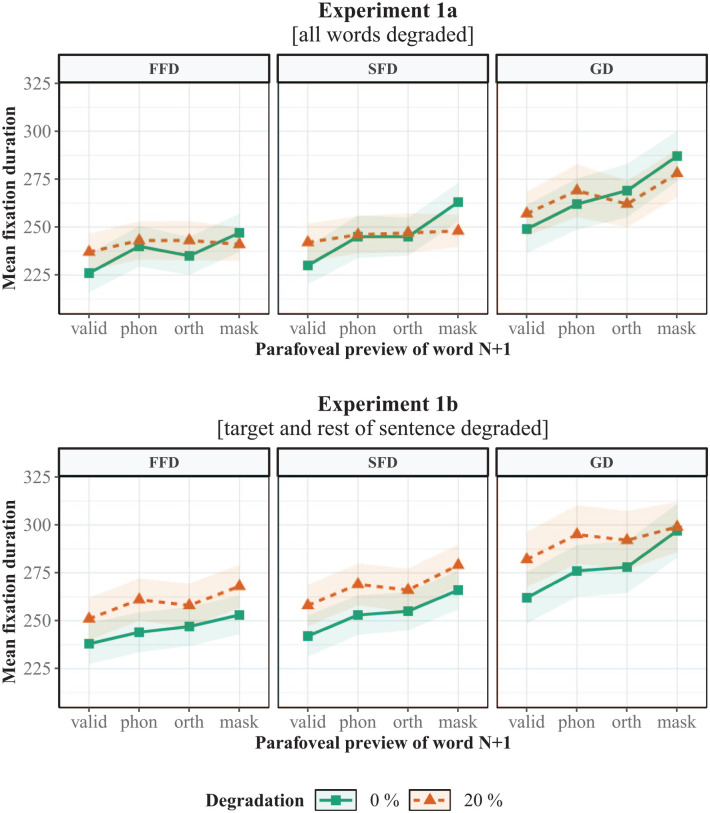
Mean fixation durations on the target word in Experiment 1a (all words degraded) and Experiment 1b (only target and rest of sentence degraded). FFD: first fixation duration; SFD: single-fixation duration; GD: gaze duration; valid: valid preview; Phon.: phonological preview; Orth.: orthographical preview; mask: letter-mask preview. Shading indicates ±1 *SE*.

Fixation durations on the target word are shown in Figure 3 and the LMM results are presented in Table 1. There was a significant invalid preview effect in all three measures, which was due to longer fixation durations following invalid letter-mask previews compared to valid previews. The orthographic preview effect was also significant in all measures, indicating that fixation durations were shorter after orthographic compared to letter-mask previews. In addition, there was a main effect of degradation in FFD, which was caused by longer FFDs following degraded compared to non-degraded previews. As predicted, there was a significant interaction between invalid preview effect and degradation in all measures. This was due to an increase in fixations durations when valid previews were degraded and a decrease in fixation durations when letter-mask previews were degraded (see Figure 3). As expected, this was due to a decrease in preview benefit in the valid preview condition and a decrease in preview cost in the invalid preview condition. In other words, the invalid preview effect was greater in the non-degraded compared the degraded condition because it also included preview costs from the letter mask. The magnitude of the preview cost was 6 ms for FFD, 15 ms for SFD, and 9 ms for GD.

**Table 1.** LMM results for fixation durations on the target word in Experiment 1a.

Fixed effects	FFD			SFD			GD		
	*b*	*SE*	*t*	*b*	*SE*	*t*	*b*	*SE*	*t*
Intercept	5.43	0.01	**388.3**	5.46	0.02	**341.5**	5.52	0.02	**328.3**
Invalid prev.	0.06	0.01	**4.43**	0.09	0.01	**6.22**	0.12	0.02	**7.5**
Orth. prev.	0.03	0.01	**2.11**	0.05	0.02	**3.54**	0.07	0.02	**4.5**
Phon. prev.	−0.01	0.01	−.71	<−0.01	0.01	−0.14	<0.01	0.02	0.17
Deg	−0.01	0.01	**−2.14**	<−0.01	0.01	−0.54	<−0.01	0.01	−0.54
Invalid prev. × Deg.	0.04	0.01	**2.99**	0.05	0.01	**3.43**	0.04	0.02	**2.58**
Orth. prev. × Deg.	0.03	0.01	**1.99**	0.03	0.02	**1.96**	0.01	0.02	0.75
Phon. prev. × Deg.	−0.01	0.01	−0.7	<−0.01	0.01	−.1	0.02	0.02	1.25
Random effects	Var.	*SD*	Corr.	Var.	*SD*	Corr.	Var.	*SD*	Corr.
Intercept (items)	0.0045	0.0675		0.0063	0.0798		0.0090	0.0951	
Intercept (subj.)	0.0072	0.0849		0.0093	0.0965		0.0087	0.0935	
Deg (subj.)	0.0004	0.0206	0.58	0.0007	0.0275	0.37	0.0006	0.0246	0.12
Residual	0.0801	0.2828		0.0720	0.2683		0.1025	0.3202	

Invalid prev.: invalid preview effect (letter mask vs. valid preview); Orth. prev.: orthographic preview effect (orthographic vs. letter-mask preview); phon. prev.: phonological preview effect (phonological vs. orthographic preview); Deg.: preview degradation; FFD: first fixation duration; SFD: single-fixation duration; GD: gaze duration; Subj.: subjects; *SE*: standard error; *SD*: standard deviation.

Statistically significant *t*-values are formatted in bold.

The interaction between orthographic preview and degradation was also significant for FFD and SFD. This reflected a similar relationship: fixation durations increased when the orthographic preview was degraded (reduction in orthographic benefit) and decreased when the letter mask was degraded (reduction in cost). In other words, the orthographic preview effect was larger in the non-degraded compared to the degraded preview condition. Nevertheless, the reduction in orthographic benefit was negligible in SFD, so the interaction was mostly driven by the reduction in cost from the letter mask. Interestingly, however, there was no main effect of phonological preview (i.e., shorter fixation durations following phonological previews compared to orthographic control previews) or an interaction between phonological preview and degradation.

#### Global reading behaviour

Because all words were visually degraded prior to their fixation in the degraded preview condition, it is important to consider whether this manipulation may have influenced participants’ global reading behaviour. The descriptive statistics for global reading measures are presented in Table 2. Sentence reading times were significantly longer in the degraded compared to the non-degraded condition (*b* = −0.03, *SE* = 0.01, *t* = −5.16). This was due to participants making more (*b* = −0.54, *SE* = 0.1, *t* = −5.76) and longer fixation durations (*b* = −0.01, *SE* = 0.002, *t* = −4.43) in the degraded compared to the non-degraded condition. There was no significant difference in saccade length between the two conditions (*b* = 0.03, *SE* = 0.03, *t* = 1.15). These results suggest that the degraded condition led to a mild slowing down of the reading process that was characterised by making more fixations and longer fixation durations.

**Table 2.** Mean descriptive statistics for global reading measures as a function of degradation condition in Experiments 1 and 2 (*SD*s in parenthesis).

Preview degradation condition	Measure			
	Sentence reading time (in ms)	Fixation duration (in ms)	Number of fixations	Saccade length (in letters)
Experiment 1a **(**all words degraded)				
Degraded (20%)	4,300 (1,889)	211 (77)	15.1 (6.33)	8.92 (8.19)
Not degraded	3,990 (1,646)	208 (79)	14 (5.56)	8.97 (8.03)
Experiment 1b (target and rest of sentence degraded)				
Degraded (20%)	4,070 (1,759)	218 (85)	13.8 (5.4)	8.95 (7.87)
Not degraded	3,880 (1,606)	216 (83)	13.3 (5.1)	8.85 (7.54)
Experiment 2a (all words degraded)				
Degraded (20%)	3,650 (1,284)	223 (86)	17.2 (3.70)	9.09 (6.96)
Not degraded	3,510 (1,400)	222 (88)	16.9 (3.73)	9.25 (6.99)
Experiment 2b **(**target and rest of sentence degraded)				
Degraded (20%)	3,760 (1,584)	228 (92)	17.2 (4.05)	9.26 (7.47)
Not degraded	3,760 (1,581)	227 (91)	17.1 (4.04)	9.15 (7.46)

In Experiments 1a and 2a, all words were degraded in parafoveal vision before they were fixated; in Experiments 1b and 2b, only the target word and the remaining sentence were degraded in parafoveal vision.

### Discussion

Experiment 1a tested whether a reliable reduction in preview costs can be observed if participants are not aware of distinct degradation at the target word location. This was achieved by degrading all words in the sentence prior to their fixation, thus ensuring that participants did not see any changes that were unique to the target word itself. Consistent with our prediction, there was evidence for a reduction in preview costs, which was shown by a decrease in fixation durations following degraded compared to non-degraded letter-mask previews. Therefore, the present results suggest that the failure to observe a reduction in preview costs on the target word in adult readers (Hutzler et al., 2019, Experiment 4; Vasilev et al., 2018) may stem from the perception of distinct degraded display changes when the manipulation is not “hidden.”

In addition, Experiment 1a replicated previous research by showing that degrading the valid preview leads to an increase in fixation durations due to a reduction in the amount of benefit that can be obtained (Findelsberger et al., 2019; Gagl et al., 2014; Hutzler et al., 2019; Marx et al., 2015, 2016, 2017). Experiment 1a also extended this result to orthographically related previews by showing that FFD increased when the orthographic previews were degraded. Similar to valid previews, this suggests that there was a reduction in the net benefit from orthographic previews. However, as this finding was not present in the remaining two dependent measures, more research is needed to understand how degradation may prevent the acquisition of orthographic information.

Interestingly, while Experiment 1a found evidence for both invalid preview effects and orthographic preview effects, the phonological preview effect did not reach significance in any of the measures. While this is contrary to some studies showing the existence of the effect (Blythe et al., 2018; Chace et al., 2005; Leinenger, 2019; Miellet & Sparrow, 2004; Pollatsek et al., 1992), the numerical difference was still in the expected direction, at least for GDs. This is consistent with a recent meta-analysis showing that the phonological preview effect in English is relatively small (approx. 5 ms) and is mostly constrained to GDs (Vasilev, Yates, et al., 2019).

Finally, there was also a mild change in global reading behaviour in the degraded preview condition. This occurred because sentence reading time was approximately 300 ms longer in the degraded compared to the non-degraded condition. The difference was due to a minor (3 ms) increase in fixation durations and participants making, on average, one more fixation per trial. This slowdown may occur, at least in part, due to participants’ inability to parafoveally pre-process upcoming words. Because all words were degraded prior to their fixation, pre-processing was limited by the added visual noise to the valid preview of these words. Therefore, participants may have made longer fixation durations to compensate for the reduced benefit.

## Experiment 1b

**Figure 4.**
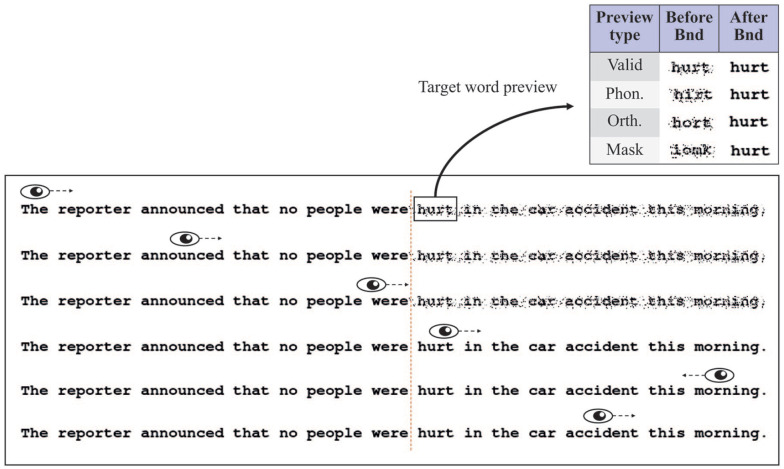
An illustration of the gaze-contingent manipulation when only the target word and the remaining sentence are degraded (Experiments 1b and 2b). Only the target word (“hurt”) and all subsequent words were degraded. Once the target word boundary was crossed, all degraded words changed to non-degraded. In the non-degraded sentence condition, reading was the same as in Experiment 1a (see Figure 2b). Note that the phonological preview condition was removed from Experiment 2b.

Experiment 1a suggested that, by eliminating the distinctiveness of target word degradation, it is possible to reliably reduce the preview costs associated with parafoveal masks. Nevertheless, Experiment 1a does not provide direct evidence that previous studies (Hutzler et al., 2019, Experiment 4; Vasilev et al., 2018) may have failed to find a decrease in preview costs due to the presence of such distinct target word degradation. To test this directly, we repeated Experiment 1a, but this time without hiding the degraded manipulation on the target word. Experiment 1b used the original incremental boundary technique (Marx et al., 2015) where only the target word and the remaining sentence were degraded (see Figure 4). This is identical to the manipulation used in previous studies (Findelsberger et al., 2019; Hutzler et al., 2019; Marx et al., 2015, 2016, 2017; Vasilev et al., 2018). Critically, the parafoveal degradation on the target word in this manipulation is visually distinct and participants are often aware of it (Findelsberger et al., 2019; Vasilev et al., 2018).

We predicted that there will be no reduction in preview costs due to the high awareness of distinct degradation at the target word location which coincides with the location of the display-change boundary. In addition, consistent with the results of Vasilev et al. (2018), we predicted that degraded previews will introduce “distinct degradation” costs, which would lead to a general increase in fixation durations (see Figure 1b). If this is indeed due to the fact that participants perceived unexpected, distinct degradation on the target word, the increase in fixation durations should occur not only in letter-mask previews, but also in phonologically and orthographically related previews. Therefore, we predicted a main effect of degradation (due to the introduction of distinct degradation costs), but no interaction with any of the three preview effects.

### Method

#### Participants

Sixty-four Bournemouth University students participated for course credit or a payment of £5 (49 female).^3^ None of them had participated in Experiment 1a. Their mean age was 20.3 years (*SD* = 2.33 years; range = 18–28 years). The study was approved by the Bournemouth University Research Ethics Committee (protocol No. 17135). All participants provided informed written consent.

#### Materials, apparatus, and procedure

The method was the same as Experiment 1a, except for the following differences. There was only one boundary located before the target word (as in the classical boundary paradigm; Rayner, 1975). The four target word preview conditions (valid, orthographic, phonological, and letter mask) were identical to Experiment 1a. However, in the degraded conditions, only the parafoveal preview of the target word and all words following the target word were degraded. Therefore, unlike Experiment 1a, all words prior to the target word were *not* degraded and the degradation occurring on the target word was no longer “hidden.” Once the target word boundary was crossed, all degraded words changed to non-degraded (see Figure 4 for an illustration). This manipulation is identical to previous studies (Marx et al., 2015, 2016, 2017; Vasilev et al., 2018). Participants were not told anything about the manipulation before the experiment and were simply asked to read for comprehension. Display changes were completed on average within 6.26 ms of the eye crossing the invisible boundary (*SD* = 1.17 ms). The statistical models had a similar random-effects structure: all models had a random intercept for items and subjects; the FFD, GD, sentence reading time, and fixation number models had Degradation random slope for subjects; SFD had a Degradation random slope for items. The models did not converge with any other random slopes.

### Results

All participants had comprehension accuracy greater than 81.2% (*M* = 93.8%; *SD* = 21.3%). Valid previews (*M* = 91.8%; *SD* = 27.5%) led to significantly lower comprehension accuracy compared to invalid letter-mask previews (*M* = 95.1%; *SD* = 21.6%), *z* = 2.22,^4^ but there were no other differences in comprehension accuracy (all |*z*|s ⩽ 1.2). When asked after the experiment, 85.9% of all participants reported noticing degraded parafoveal previews and 23.4% of all participants reported noticing non-degraded letter-mask previews or single-letter changes (in the non-degraded phonological or orthographic preview conditions). The fixation data was pre-processed in the same way as Experiment 1a. After the pre-processing stage, 73.7 % of the data was left for analysis (13.4 % was removed due to blinks or track losses, 12.7 % due to late or inappropriate triggering of the target word boundary, and 0.14 % was removed as outliers).

#### Target word

Mean fixation durations on the target word in Experiment 1b are presented in Figure 3 and the LMM results are shown in Table 3. Similar to Experiment 1a, the invalid preview effect was significant in all three measures. This was due to shorter fixation durations following valid compared to letter-mask previews. The orthographic preview effect was also significant in all measures. In addition, there was a robust main effect of degradation, which indicates that degraded previews resulted in longer fixation durations compared to non-degraded previews. Similar to Experiment 1a, there was no significant phonological preview effect in any of the measures. The only significant interaction in the experiment was between the invalid preview effect and degradation in GD. This was due to an increase in GD when valid previews were degraded (reduction in benefit), but no such increase occurred when the letter masks were degraded. In other words, the invalid preview effect was larger in the non-degraded compared to the degraded condition. This occurred because, unlike FFD and SFD, degradation did not result in an increase in GD for letter-mask previews. However, this still does not indicate a reduction in preview cost similar to the one observed in Experiment 1a, as GD would need to be shorter compared to the non-degraded letter-mask preview. Finally, when the target word data from Experiment 1a and 1b were combined, degradation resulted in significantly longer fixation durations in Experiment 1b compared to Experiment 1a (see Supplementary Material 1).

**Table 3.** LMM results for fixation durations on the target word in Experiment 1b.

Fixed effects	FFD			SFD			GD		
	*B*	*SE*	*t*	*b*	*SE*	*t*	*b*	*SE*	*t*
Intercept	5.48	.02	**319.5**	5.52	.02	**297.5**	5.58	.02	**279.1**
Invalid prev.	.07	.01	**4.81**	.1	.02	**6.38**	.11	.02	**6.59**
Orth. prev.	.03	.01	**2.11**	.04	.02	**2.7**	.05	.02	**2.99**
Phon. prev.	.01	.01	.39	.01	.02	.51	.01	.02	.46
Deg.	−.03	.01	**−4.43**	−.03	.01	**−4.76**	−.03	.01	**−3.85**
Invalid prev. × Deg.	<.01	.01	.3	.02	.02	1.22	.04	.02	**2.32**
Orth. prev. × Deg	−.01	.01	−.67	<−.01	.02	−.01	.01	.02	.76
Phon. prev. × Deg	.02	.01	1.49	.01	.02	.81	.02	.02	1.19
Random effects	Var.	*SD*	Corr.	Var.	*SD*	Corr.	Var.	*SD*	Corr.
Intercept (items)	.0031	.0557		.0049	.0706		.0059	.0769	
Deg. (items)				.0003	.0188	−.17			
Intercept (subj.)	.0145	.1207		.0159	.1261		.0187	.1367	
Deg (subj.)	.0007	.0271	−.43				.0006	.0253	−.34
Residual	.0874	.2956		.0787	.2805		.1090	.3302	

Invalid prev.: invalid preview effect (letter mask vs. valid preview); orth. prev.: orthographic preview effect (orthographic vs. letter-mask preview); phon. prev.: phonological preview effect (phonological vs. orthographic preview); Deg.: preview degradation; FFD: first fixation duration; SFD: single-fixation duration; GD: gaze duration; subj.: subjects; *SE*: standard error; *SD*: standard deviation.

Statistically significant *t*-values are formatted in bold.

#### Global reading behaviour

The descriptive statistics for global reading measures are presented in Table 2. Similar to Experiment 1a, sentence reading time was significantly longer in the degraded compared to the non-degraded preview condition (*b* = −0.02, *SE* = 0.01, *t* = −4.26). The degraded condition also led to more fixations (*b* = −0.24, *SE* = 0.07, *t* = −3.29) and longer fixation durations (*b* = −0.003, *SE* = 0.001, *t* = −2.48) compared to the non-degraded condition. There was again no significant difference in saccade length between the two conditions (*b* = −0.04, *SE* = 0.03, *t* = −1.41). In summary, the results from global reading measures replicate the findings from Experiment 1a and suggest that there is a mild change in global reading behaviour even when the visual degradation is administered with the original incremental boundary paradigm also used in previous studies (Marx et al., 2015, 2016, 2017; Vasilev et al., 2018).

### Discussion

Experiment 1b tested whether a reduction in preview costs can be observed when readers perceive distinct target word degradation. In contrast to Experiment 1a where the manipulation was hidden, there was no evidence for a reduction in preview costs in Experiment 1b. Rather, degradation led to a general increase in fixation durations, which was particularly evident in earlier measures such as FFD and SFD. Degradation also resulted in significantly longer fixation durations in Experiment 1b compared to Experiment 1a (see Supplementary Material 1). This suggests that, instead of decreasing the costs associated with invalid previews, degradation introduced additional costs of its own. Similar to Experiment 1a, there was no evidence for the phonological preview effect, although the effects sizes were again in the expected direction and comparable to recent estimates (Vasilev, Yates, et al., 2019). The lack of this effect in the two experiments is not necessarily a problem as the present research was mainly interested in how orthographic and phonological previews are influenced by degradation.

Interestingly, there was no increase in GD in the degraded letter-mask condition. This is in agreement with the results from Vasilev et al.’s (2018) Experiment 1 and suggests that the cost of distinct degradation changes is largely constrained to the first fixation on the target word. This may occur if degradation delays the linguistic processing of the target word immediately after crossing the boundary due to its perceptual salience. Because degradation disappears once the boundary is crossed, it may no longer affect subsequent re-fixations (which contribute towards GD).

A further interesting finding from Experiment 1b was that degradation led to a very similar increase in fixation durations in the orthographic and phonological preview conditions. Because these previews were much more “word-like” than the letter masks, the present results suggest that the presence of distinct target word degradation leads to an increase in fixation durations that is largely independent of type of preview that is used. The orthographic and phonological previews in the present research differed from the valid preview only by a single letter. Therefore, there is hardly any letter “masking” in the way that can potentially introduce preview costs. Rather, any increase in fixation durations in the degraded condition can likely be attributed to the presence of distinct target word degradation. The significance of these results is that the added cost of degradation when the manipulation is not hidden may conceal any decreases in preview costs from the letter mask that were observed in Experiment 1a.

Finally, degradation influenced global reading measures in a similar way to Experiment 1a. While the mean difference was less pronounced, participants still spent more time reading the sentences in the degraded compared to the non-degraded condition, which was again caused by slightly more and longer fixations. However, unlike Experiment 1a, the degradation was limited to a single-display change on the target word and it disappeared after this word was fixated. Therefore, the mild slowdown of reading processes may be due to participants’ noticing the distinct parafoveal degradation prior to crossing the target word boundary. This is because the degraded post-target words would likely fall too far in peripheral vision for any meaningful parafoveal pre-processing to occur (see Rayner, 1998; Schotter et al., 2012).

## Experiment 2

To evaluate the robustness of the key findings from Experiment 1, a high-power replication was conducted. Because recent evidence has suggested that the phonological preview effect in adult English readers may be smaller than originally thought (Vasilev, Yates, et al., 2019), the phonological preview condition was removed.^5^ This does not affect the research aims of the original experiment as the phonological and orthographic previews are complementary in the research design (i.e., when target word degradation is “hidden” by degrading all words in the sentence, this degradation should reduce the amount of orthographic and phonological benefit that readers obtain parafoveally). Therefore, due to its larger size, the orthographic preview effect is a better candidate for testing this prediction. In this sense, Experiment 2 was a direct, high-power replication of Experiment 1, but without the phonological preview condition.

### General method

The method of Experiment 2a was identical to that of Experiment 1a and the method of Experiment 2b was identical to that of Experiment 1b, except for the following differences.

#### Participants

One hundred and twenty Bournemouth University students took part for course credit (60 in Experiment 2a [50 female] and 60 in Experiment 2b [50 female]). Their average age was 19.6 years in Experiment 2a (*SD* = 2.16 years; range = 18–32 years) and 20.4 years in Experiment 2b (*SD* = 4.97 years; range = 18–53 years). None of them had participated in the previous experiments. One more participant was tested in Experiment 2a and three more in Experiment 2b, but they had to be replaced due to poor tracking. Both studies were approved by the Bournemouth University Research Ethics Committee (protocol No. 28816) and all participants provided informed written consent. Prospective statistical power was calculated with the *simr* R package v.1.0.5 (Green & Macleod, 2016) based on the data from Experiment 1. With 60 participants, Experiment 2a had an average power of 0.964 (*SD* = 0.057; range 0.822–0.999), and Experiment 2b had an average power of 0.991 of detecting the predicted effects (*SD* = 0.022; range = 0.932–0.999) (for more details, see Supplementary Material 2).

#### Materials and design

After the removal of the phonological preview condition, Experiment 2 had a 2 (degradation: 0 vs. 20%) × 3 (target word preview: valid, orthographic, letter mask) within-subject design. The design was otherwise identical to that of Experiment 1: in Experiment 2a all words were degraded prior to their fixation, whereas in Experiment 2b only the target word and remaining sentence were degraded.

The 80 items from Experiment 1 were combined with 58 new items written for Experiment 2, thus bringing the total number of items to 138 (see Supplementary Material 2). The new items were written in the same way as the original ones from Experiment 1. In the complete corpus of 138 items, the target word was 4.77 letters on average (*SD* = 0.61 letters; range = 4–6 letters) and the pre-target word was 5.65 letters on average (*SD* = 1.27 letters; range = 4–8 letters). The sentences were 14.17 words long on average (*SD* = 1.73 words; range: 9–18 words) and the mean target word position in the sentence was 7.49 (*SD* = 2.15 words; range = 4–14 words).

#### Apparatus and procedure

The experiments were conducted in a different room, but with a similar layout to the one used in Experiment 1. Eye-movements were recorded with an EyeLink 1000 Plus eye-tracker at 1,000 Hz. The sentences were presented on a Lacie Electron 22 Blue IV CRT monitor (resolution: 1,024 × 768; refresh rate: 150 Hz). The letter width was 11 px and the eye-to-monitor distance was 70 cm. Each letter subtended ~0.35 º horizontally. The experiments were programmed in Matlab R2014a (MathWorks, 2014) using the Psychophysics Toolbox v.3.0.15 (Brainard, 1997; Pelli, 1997) and EyeLink libraries (Cornelissen et al., 2002). The experiments were run on a Windows 7 computer. Display changes were completed on average within 8.72 ms of the eye crossing the boundary (*SD* = 3.59 ms) in Experiment 2a and 8.31 ms in Experiment 2b (*SD* = 1.95 ms). Participants pressed the left mouse button to terminate trials and to answer the comprehension questions. The experiments took about 40 minutes to complete. The LMM models converged only with a Degradation slope for subjects or items (or both), except for the SFD model in Experiment 2a, which converged only with a subject slope for Parafoveal Preview. The exact random-effects structure for each model is reported in Tables 4 and 5.

**Table 4.** LMM results for fixation durations on the target word in Experiment 2a (replication of Experiment 1a).

Fixed effects	FFD			SFD				GD		
	*b*	*SE*	*t*	*b*	*SE*	*t*		*b*	*SE*	*t*
Intercept	5.51	.02	**351.6**	5.53	.02	**338.4**		5.57	.02	**322.8**
Invalid prev.	.10	.01	**9.13**	.11	.01	**8.63**		.12	.01	**10.9**
Orth. prev.	.04	.01	**3.53**	.05	.01	**4.23**		.06	.01	**5.84**
Deg	<−.01	.01	−.28	<−.01	<.01	−.6		<.01	<.01	.64
Invalid prev. × Deg	.05	.01	**4.82**	.06	.01	**5.53**		.06	.01	**5.14**
Orth. prev. × Deg	.03	.01	**2.56**	.03	.01	**3.02**		.04	.01	**3.37**
Random effects	Var.	*SD*	Corr.	Var.	*SD*	Corr.		Var.	*SD*	Corr.
Intercept (items)	.0043	.0656		.0052	.0726			.0074	.0863	
Intercept (subj.)	.0116	.1080		.0124	.1117			.0133	.1154	
Deg. (subj.)	.0004	.0215	−.38					.0002	.0148	−.03
Invalid prev. (subj.)				.0032	.0574	.16				
Orth. prev. (subj.)				.0015	.0393	.08	.81			
Residual	.0929	.3048		.0884	.2973			.1018	.3191	

Invalid prev.: Invalid preview effect (letter mask vs. valid preview); Orth. prev.: orthographic preview effect (orthographic vs. letter-mask preview); Deg.: preview degradation; FFD: first fixation duration; SFD: single-fixation duration; GD: gaze duration; Subj.: subjects; *SE*: standard error; *SD*: standard deviation.

Statistically significant *t*-values are formatted in bold.

**Table 5.** LMM results for fixation durations on the target word in Experiment 2b (replication of Experiment 1b).

Fixed effects	FFD			SFD			GD		
	*b*	*SE*	*t*	*b*	*SE*	*t*	*b*	*SE*	*t*
Intercept	5.55	.02	**331.3**	5.57	.02	**320.3**	5.61	0.02	**323.1**
Invalid prev.	.10	.01	**9.75**	.12	.01	**10.93**	0.12	0.01	**10.73**
Orth. prev.	.06	.01	**5.79**	.07	.01	**6.19**	0.07	0.01	**6.48**
Deg.	−.04	.01	−**8.52**	−.05	.01	−**8.44**	−0.04	0.01	−**8.14**
Invalid prev. × Deg	.04	.01	**3.42**	.04	.01	**3.76**	0.05	0.01	**4.36**
Orth. prev. × Deg	.02	.01	**2.35**	.02	.01	**2.07**	0.02	0.01	**2.1**
Random effects	Var.	*SD*	Corr.	Var.	*SD*	Corr.	Var.	*SD*	Corr.
Intercept (items)	.0036	.0603		.0039	.0626		.0054	.0733	
Deg. (items)	.0006	.0237	−.20	.0005	.0219	−.08			
Intercept (subj.)	.0140	.1184		.0151	.1230		.0144	.120	
Deg. (subj.)	.0002	.0152	−.37	.0004	.0188		.0005	.0220	−.33
Residual	.0902	.3003		.0861	.2935		.1021	.3196	

Invalid prev.: Invalid preview effect (letter mask vs. valid preview); Orth. prev: orthographic preview effect (orthographic vs. letter-mask preview); Deg.: preview degradation; FFD: first fixation duration; SFD: single-fixation duration; GD: gaze duration; Subj.: subjects; *SE*: standard error; *SD*: standard deviation.

Statistically significant *t*-values are formatted in bold.

### Results

#### Experiment 2a

Comprehension accuracy was 96.1% on average (*SD* = 2.87%; range: 89%–100%). Comprehension accuracy was significantly higher in the letter mask (*M* = 96.9%; *SD* = 17.2%) compared to the valid preview condition (*M* = 95.5%; *SD* = 20.8%), *z* = 2.01. There were no other significant differences in accuracy (all |*z*|s ⩽ 1.49). All participants noticed the degraded display changes and 23.3% of participants noticed letter display changes in the non-degraded condition. During pre-processing, 10.82% of trials were removed due to blinks, 13.65% due to late or inappropriate boundary triggering, and 0.1% due to outliers. This left 75.43% of the data for analysis.

**Figure 5.**
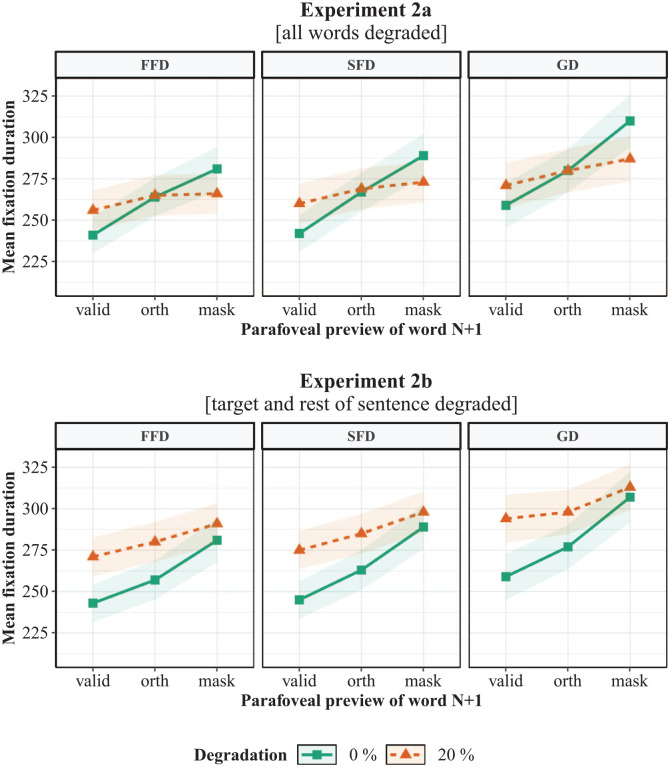
Mean fixation durations on the target word in Experiment 2a (all words degraded) and Experiment 2b (only target and rest of sentence degraded). Experiment 2a was a replication of Experiment 1a and Experiment 2b was a replication of Experiment 1b, without the phonological preview condition. FFD: first fixation duration; SFD: single-fixation duration; GD: gaze duration; valid: valid preview; orth: orthographical preview; mask: letter-mask preview. Shading indicates ±1 *SE*.

Mean fixation durations on the target word are illustrated in Figure 5 and the LMM results are shown in Table 4. Consistent with Experiment 1a, the main effects of invalid preview and orthographic preview were both significant in all measures. This indicates that letter-mask previews led to longer fixation durations compared to valid previews and that orthographic previews led to shorter fixation durations compared to letter-mask previews. In addition, the main effect of degradation was not significant in any of the measures. Importantly, the interaction between the invalid preview effect and degradation was significant, also replicating Experiment 1a. As Figure 5 shows, this occurred because degrading the valid preview led to an increase in fixation durations (i.e., reduction in benefit), whereas degrading the letter mask resulted in a decrease in fixation durations (i.e., reduction in cost). Finally, the interaction between the orthographic preview effect and degradation was also significant. Similar to above, this occurred because fixations durations decreased when the letter mask was degraded (reduction in cost), but there was a minor increase in fixation durations when the orthographic preview was degraded (reduction in orthographic benefit). However, consistent with Experiment 1a, the reduction in orthographic benefit was only marginal.

Descriptive statistics for global reading measures in Experiment 2a are presented in Table 2. Similar to Experiment 1a, the degraded condition led to a significant increase in sentence reading time compared to the non-degraded condition (*b* = −0.03, *SE* = 0.005, *t* = −4.92). This was again due to longer fixation durations (*b* = −0.005, *SE* = 0.001, *t* = −4.83) and more fixations per trial (*b* = −0.12, *SE* = 0.04, *t* = −3.09) in the degraded compared to the non-degraded condition. However, unlike Experiment 1a, the degraded condition also led to slightly shorter saccade lengths compared to the non-degraded condition (*b* = 0.078, *SE* = 0.02215, *t* = 3.52).

#### Experiment 2b

Comprehension accuracy was 96.2% on average (*SD* = 2.9%; range: 87%–100%), and there were no significant differences across the conditions (all |*z*|s ⩽ 1.37). When asked after the study, 93.3% of participants reported seeing degraded display changes and 46.6% reported noticing letter changes in the non-degraded condition. During pre-processing, 9.49% of trials were removed due to blinks, 17.2% due to late or inappropriate boundary triggering, and 0.38% due to outliers. This left 72.9% of the data for analysis.

Target word fixation durations are illustrated in Figure 5, and the LMM results are shown in Table 5. Consistent with Experiment 1b, there were significant invalid preview and orthographic preview effects. These were again due to longer fixation durations following letter mask compared to valid previews, and shorter fixation durations following orthographic compared to letter-mask previews. In addition, there was a robust main effect of degradation, also replicating Experiment 1b. This was due to a general increase in fixation durations in the degraded compared to the non-degraded condition. However, unlike Experiment 1b, the interaction between invalid preview effect and degradation, and orthographic preview and degradation was significant in all measures. As Figure 5 shows, both interactions were driven by the fact that the increase in fixation durations with degradation in the letter-mask preview was smaller compared to the valid and orthographic preview, respectively. Therefore, while degradation led to an increase in fixation durations in all preview conditions, this increase was smaller in the letter-mask condition.

Mean global reading measures are reported in Table 2. Unlike Experiment 1b, reading times did not significantly differ between the degraded and non-degraded condition (*b* = −0.001, *SE* = 0.003, *t* = −0.38). While the number of fixations per trial also did not differ between the degraded and non-degraded condition (*b* = −0.018, *SE* = 0.038, *t* = −0.46), participants made slightly longer fixations (*b* = −0.003, *SE* = 0.001, *t* = −2.13) and saccades (*b* = −0.05, *SE* = 0.024, *t* = −2.18) in the degraded compared to the non-degraded condition.

### Discussion

Experiment 2 replicated the key findings from Experiment 1. When all words were parafoveally degraded in Experiment 2a, thus removing the distinctiveness of target word degradation, we obtained the predicted cross-over in the invalid preview effect. Specifically, degrading the valid preview led to an increase in fixation durations (reduction in preview benefit), while degrading the letter-mask preview led to a decrease in fixation durations (reduction in preview cost). A similar pattern was also observed for the orthographic preview effect, although the reduction in orthographic benefit was only marginal. Critically, however, when only the target word and remaining sentence were degraded in Experiment 2b, as in the original incremental boundary paradigm (Marx et al., 2015), this pattern was no longer there. Instead, there was a general increase in fixation durations with degraded previews and there was no evidence for a reduction in preview costs. A comparison between the two experiments again indicated that degradation led to significantly longer fixation durations in Experiment 2b compared to Experiment 2a (see Supplementary Material 1).

Admittedly, the increase in fixation durations in the degraded condition was somewhat smaller in the letter mask compared to the valid and orthographic preview conditions. However, in no case were fixation durations shorter in the degraded letter-mask condition compared to the non-degraded letter-mask condition. As such, there was again no evidence for a reduction in preview costs in the original version of the incremental boundary paradigm (Marx et al., 2015). Therefore, consistent with Experiment 1, preview costs were reduced only in the modified version of the paradigm where the distinctiveness of target word degradation was removed by degrading all words in the sentence.

Finally, consistent with Experiment 1a, parafoveal degradation in Experiment 2a again led to longer sentence reading times, which was due to participants making slightly more and slightly longer fixations. However, this was generally not the case in Experiment 2b where only the target and remaining sentence were degraded (except for fixations still being slightly longer in the degraded condition). In addition, saccade lengths were also influenced by degradation in both Experiment 2a and 2b, although the mean difference was very small. In summary, reading times were longer when all words were degraded in the sentence, but degradation otherwise had only a minor effect on global reading behaviour.

## General discussion

The present research investigated how the distinctiveness of target word degradation influences preview benefits and preview costs during reading. The results showed that the preview costs associated with invalid masks can be reduced when participants do not perceive distinct parafoveal degradation at the target word location (Experiments 1a and 2a), but that no such reduction in preview costs occurs when such distinct degradation is present (Experiments 1b and 2b). This was because the distinct target word degradation in Experiments 1b and 2b led to additional costs that inflated fixation durations and likely concealed the desired reduction in preview cost from the mask. In addition, this increase in fixation durations that occurred with distinct degradation changes was very similar for phonologically and orthographically related previews. This suggests that the cost associated with the presence of distinct target word degradation changes is not specific to invalid masks, but also affects previews that contain some information about the target word.

The reduction in preview costs in Experiments 1a–2a replicates Marx et al.’s (2015) original findings and provides further evidence that invalid masks may inflate the preview effect (Hutzler et al., 2019; Marx et al., 2016, 2017). Critically, however, the present data also demonstrate that the use of parafoveal degradation in such studies has the unintended consequence of adding additional costs due to the presence of highly distinct changes at the target word location (see Vasilev et al., 2018; Experiment 2). This may explain why some studies (Hutzler et al., 2019, Experiment 4; Vasilev et al., 2018, Experiment 1) have failed to replicate the decrease in preview costs on the target word that Marx et al. (2015) originally reported. In this sense, the present data show that the incremental boundary technique (Marx et al., 2015) can be a very useful tool for studying the preview costs associated with invalid masks. However, it is important to eliminate the distinctiveness of degraded display changes on the target word when using this technique.

It is worth noting that the additional cost associated with the awareness of distinct target word degradation in the original incremental boundary paradigm was either absent (Experiment 1b) or less pronounced (Experiment 2b) in GD for the letter-mask condition. This is consistent with the results from Vasilev et al.’s (2018) Experiment 1, where the additional cost for letter masks (i.e., longer fixation durations when masks are degraded) was present for FFD, but not GD. This may occur because degradation disappears once the target word boundary is crossed. Therefore, the degradation cost may be largely constrained to the first post-boundary fixation on the target word. During re-fixations (which count towards GD), the degradation was no longer present on the previous fixation, so this additional cost may get diluted and begin to disappear. GD is moderately correlated with FFD (von der Malsburg & Angele, 2017) because the two measures are the same when the target is not re-fixated. In these cases, any degradation cost originating from FFD will also be carried over to GD. However, if target word re-fixations are no longer influenced by degradation because the preview has already disappeared, the cost will get diluted because the “unique” information in GD (i.e., the re-fixations) will remain unaffected by degradation. Therefore, GD may be a mixture of cases where there is cost (carried over from FFD) and cases where there is little cost due to the added re-fixations. This would be in line with the results from Experiment 2b where the cost in GD decreased compared to FFD, but was still somewhat present.

The advantage of the present studies and those by Marx et al. (2015) and Hutzler et al. (2019) is that they experimentally manipulate the extent to which invalid masks can cause interference, thus making it possible to study preview costs directly. This is an improvement over other studies that have used naturally occurring variation in landing positions (Kliegl et al., 2013) or observational data (Vasilev & Angele, 2017). As a result, they provide important evidence for the existence of preview costs and their likely magnitude. Experiments 1a and 2a suggested that preview costs may account for between 23% and 45% of the size of the invalid preview effect in first-pass measures (*M* = 35.7%; *SD* = 8.7%). This is important for interpreting the results from boundary studies, as current estimates are likely to be a combination of preview benefits and preview costs, which may overestimate the size of the true benefit (Hutzler et al., 2019; Marx et al., 2015).

While the present results demonstrate the reality of preview costs, they do not tell us why they occur in the first place. One possibility first suggested by Kliegl et al. (2013) is that invalid masks cause interference (i.e., processing cost) because readers try to process them. This is a plausible explanation as letter masks are non-words that do not exist in readers’ mental lexicon. Because such masks have a lexical frequency of 0, they may lead to a “maximal” processing difficulty that interferes with word recognition (Risse et al., 2014). In addition, letter masks are often generated (pseudo)randomly, which can lead to highly irregular letter combinations that are not commonly found in natural language. Some reading models (e.g., Snell et al., 2018) assume that word recognition depends on the activation of open letter bigrams from the visual input. Therefore, if irregular bigram activations from the mask persist after readers fixate the target word, they may interfere with word recognition processes. Therefore, non-distinct degradation may act to prevent this bigram activation during parafoveal processing.

A second explanation is that the preview costs associated with invalid masks may be at least partially due to display-change awareness. This is supported by the finding that readers are more likely to notice letter changes when their gaze position is closer to the mask (Slattery, Angele, & Rayner, 2011). Incidentally, however, this is also when the mask is most likely to be processed and cause interference (Kliegl et al., 2013). Therefore, one task for future research would be to try to dissociate any interference caused by the mask from the potential awareness of letter changes. Nevertheless, the two explanations are not mutually exclusive, so preview costs may well be due to a combination of interference and the detection of letter changes. In fact, the activation of illegal letter combinations may play a role in the detection of changes (Angele et al., 2016).

The present results also showed clear costs associated with the presence of distinct target word degradation in Experiments 1b–2b. This is consistent with previous findings showing that display-change awareness can lead to inflated fixation durations (Angele et al., 2016; Vasilev et al., 2018; White et al., 2005). Currently, it is not clear whether the distinct target word degradation is perceived before or after the display change happens. The traditional interpretation of display-change awareness (e.g., Slattery, Angele, & Rayner, 2011) is that participants notice the flicker as the preview changes to the actual target word when the boundary is crossed. However, given the very high awareness of degradation in the incremental boundary paradigm, it may well be the case that participants are aware of the degradation even before the display change happens. The lack of parafoveal-on-foveal effects of degradation in the present experiments (see Supplementary Material 2) could be taken as indirect evidence that this awareness did not occur while participants were fixating on the pre-target word. Nevertheless, it cannot be excluded that participants notice the degradation in peripheral vision even before they reach the pre-target word, in which case such parafoveal-on-foveal effects may not necessarily occur. Therefore, one interesting question for future research would be establish at which point during sentence reading participants become aware of the distinct target degradation and how exactly this translates into the increase in fixation durations on the target word. Nevertheless, regardless of when this happens, the present data clearly suggest that there is a cost associated with the presence of distinct degradation at the target word location.

The present experiments had very similar manipulations, but the critical difference was that the degradation manipulation on the target word was distinct in Experiments 1b and 2b, but not in Experiments 1a and 2a where every word was degraded and the presence of degradation was “normal.” The lack of inflated target word fixation durations in Experiments 1a and 2a clearly suggests that the mere presence of salient degradation is not enough for such costs to occur. Rather, degradation also needs to be distinct in a manner that highlights the target word and display-change location, as was the case in Experiments 1b and 2b.

It is well known that perceptually novel or distinct stimuli can capture attention away from the main task by eliciting an orienting response (Sokolov, 1963, 2001). For example, unexpected sounds can lead to an involuntary switch of attention in tasks such as reading (Marois & Vachon, 2018; Vasilev, Parmentier, et al., 2019) or scene viewing (Graupner et al., 2007). Similarly, visual objects with sudden or unexpected onset can also capture attention (Brockmole & Henderson, 2005; Pereira & Castelhano, 2019; Theeuwes et al., 1998; Yantis & Jonides, 1984). Therefore, visual degradation may lead to such costs because attention is temporarily redirected away from the reading task and towards the distinct degraded stimuli. As a result, the increase in fixation durations in response to distinct degradation in Experiments 1b and 2b may be due to an attention orienting response that delays the onset of word recognition processes.

### Implications for computational models of reading

While current models of reading (e.g., Engbert et al., 2005; Reichle et al., 1998; Snell et al., 2018) can account for benefits from parafoveal previews, they do not necessarily simulate the exact sequence of events that occur in boundary-change experiments. Invalid preview conditions have been simulated in different ways, such as assuming that lexical processing does not start until the target word is fixated (Pollatsek et al., 2006; Sheridan & Reichle, 2016), that word recognition processes (i.e., lexical activations) are reset after crossing the boundary (Risse et al., 2014) or by remaining agnostic and not simulating invalid previews directly (Schotter et al., 2014). Critically, none of these methods can account for interference costs from parafoveal masks or the cost from distinct degradation changes. This in turn highlights the need for a better understanding of why these effects occur and how to model them computationally.

On the surface, these phenomena may simply be viewed as methodological artefacts that are best to be avoided. However, they also offer the opportunity to model more directly how task-irrelevant factors can influence oculomotor control during reading. The awareness costs associated with noticing the distinct degraded changes in Experiments 1b and 2b suggest that perceptually salient (i.e., distinct) stimuli can attract attention away from reading processes. Therefore, these effects can be viewed as an instance of task-irrelevant distractors that can briefly influence the allocation of attention during reading. By considering how external factors can modulate reading processes, computational models will come closer not only to explaining display-change awareness effects, but potentially also broader phenomena such as task-irrelevant auditory distractors (Cauchard et al., 2012; Hyönä & Ekholm, 2016; Vasilev, Liversedge, et al., 2019; Yan et al., 2018; Zhang et al., 2018) or the modulation of attention by task demands (Kaakinen & Hyönä, 2010; Radach et al., 2008; Schotter et al., 2014; Weiss et al., 2018; Wotschack & Kliegl, 2013).

One way to account for distinct degradation costs could be Angele et al.’s (2016) two-stage model of parafoveal processing. In this model, display-change awareness costs occur early in processing and are a function of the orthographic properties of the preview. When parafoveal previews are visually distinct (e.g., those with low bigram frequency, those with all-uppercase letters in lower case sentences, or those with distinct degradation), readers may notice them pre-attentively and begin to engage in separate, non-language processes. This may result in parafoveal-on-foveal effects (as in Angele et al., 2016) or delay the onset of word recognition of the target word following the display change (as in the present research). Lexical processing of the preview would occur in a later, attention-dependent stage. Therefore, according to this account, costs due to display-change awareness should be separable from those due to lexical interference from invalid masks. Future research can test this prediction by utilising the change-detection paradigm to isolate trials where participants noticed display changes from trials where they did not, in conjunction with the incremental boundary paradigm to isolate costs due to the activation of incorrect letters. Trials with detected changes should lead to similar fixation durations due to this awareness and should not be influenced by the orthographic or phonologic properties of the invalid preview, but only when these previews were degraded. However, non-degraded trials with no detected changes should be influenced only by the properties of the invalid preview.

## Conclusion

There is a growing understanding that the invalid preview effect may represent a mixture of preview benefits from the valid preview and preview costs due to interference from invalid masks (Hutzler et al., 2019; Kliegl et al., 2013). One way to demonstrate the existence of preview costs is to visually degrade invalid masks (Marx et al., 2015), but this manipulation has been shown to be easily noticeable by participants (Vasilev et al., 2018). The present study showed that preview costs can be reliably demonstrated, but only when participants are unaware of distinct degradation occurring on the target word. This suggests that noticing distinct degradation changes adds additional costs that lead to a general increase in fixation durations. This may occur because the distinct degradation temporarily attracts attention away from the reading task and delays word processing.

## Supplemental Material

QJE-STD-19-283.R2-Supplementary_Material – Supplemental material for Parafoveal degradation during reading reduces preview costs only when it is not perceptually distinct Click here for additional data file. Supplemental material, QJE-STD-19-283.R2-Supplementary_Material for Parafoveal degradation during reading reduces preview costs only when it is not perceptually distinct by Martin R Vasilev, Mark Yates, Ethan Prueitt and Timothy J Slattery in Quarterly Journal of Experimental Psychology

## Notes

1. This has traditionally been termed the *preview benefit* effect (Rayner, 1998). However, due to recent evidence suggesting that the effect may consist of not only benefit, but also cost, we use the more neutral term “preview effect” throughout the paper.

2. One more participant was tested but their data were discarded due to tracking problems.

3. Two more participants were tested but later excluded: one due to tracking problems and one due to low comprehension accuracy (<80%).

4. The mean difference was largely due to comprehension being lower in the non-degraded valid (*M* = 90.2%; *SD* = 29.7%) compared to the non-degraded letter-mask condition (*M* = 93.4%; *SD* = 24.9%). The degraded valid (*M* = 93.4%; *SD* = 24.9%) and degraded letter-mask (*M* = 94.9%; *SD* = 22%) conditions had more similar accuracy. An examination of the subject means indicated that the effect was largely due to three subjects who had low accuracy in the valid preview condition, but ceiling-level accuracy in the letter-mask condition.

5. Experiment 1 was conducted in 2016–2017 before this information was available.

## References

[bibr1-1747021820959661] Angele B. Slattery T. J. Rayner K. (2016). Two stages of parafoveal processing during reading: Evidence from a display change detection task. Psychonomic Bulletin & Review, 23(4), 1241–1249. 10.3758/s13423-015-0995-0 26769246 PMC4974265

[bibr2-1747021820959661] Baayen H. Davidson D. J. Bates D. M. (2008). Mixed-effects modeling with crossed random effects for subjects and items. Journal of Memory and Language, 59(4), 390–412. 10.1016/j.jml.2007.12.005

[bibr3-1747021820959661] Balota D. A. Pollatsek A. Rayner K. (1985). The interaction of contextual constraints and parafoveal visual information in reading. Cognitive Psychology, 17(3), 364–390. 10.1016/0010-0285(85)90013-1 4053565

[bibr4-1747021820959661] Barr D. J. Levy R. Scheepers C. Tily H. J. (2013). Random effects structure for confirmatory hypothesis testing: Keep it maximal. Journal of Memory and Language, 68(3), 255–278. 10.1016/j.jml.2012.11.001 PMC3881361 24403724

[bibr5-1747021820959661] Bates D. M. Machler M. Bolker B. M. Walker S. C. (2014). Fitting linear mixed-effects models using lme4. Journal of Statistical Software, 67(1), 1–48. 10.18637/jss.v067.i01

[bibr6-1747021820959661] Bélanger N. N. Mayberry R. I. Rayner K. (2013). Orthographic and phonological preview benefits: Parafoveal processing in skilled and less-skilled deaf readers. Quarterly Journal of Experimental Psychology, 66(11), 2237–2252. 10.1080/17470218.2013.780085 PMC3808502 23768045

[bibr7-1747021820959661] Blythe H. I. Dickins J. H. Kennedy C. R. Liversedge S. P. (2018). Phonological processing during silent reading in teenagers who are deaf/hard of hearing: An eye movement investigation. Developmental Science, 21(5), e12643. 10.1111/desc.12643 29356239

[bibr8-1747021820959661] Brainard D. H. (1997). The Psychophysics Toolbox. Spatial Vision, 10(4), 433–436. 10.1163/156856897X00357 9176952

[bibr9-1747021820959661] Brockmole J. R. Henderson J. M. (2005). Prioritization of new objects in real-world scenes: Evidence from eye movements. Journal of Experimental Psychology: Human Perception and Performance, 31(5), 857–868. 10.1037/0096-1523.31.5.857 16262483

[bibr10-1747021820959661] Cauchard F. Cane J. E. Weger U. W. (2012). Influence of background speech and music in interrupted reading: An eye-tracking study. Applied Cognitive Psychology, 26(3), 381–390. 10.1002/acp.1837

[bibr11-1747021820959661] Chace K. H. Rayner K. Well A. D. (2005). Eye movements and phonological parafoveal preview: Effects of reading skill. Canadian Journal of Experimental Psychology / Revue Canadienne de Psychologie Expérimentale, 59(3), 209–217. 16248500 10.1037/h0087476

[bibr12-1747021820959661] Cornelissen F. W. Peters E. M. Palmer J. (2002). The Eyelink Toolbox: Eye tracking with MATLAB and the Psychophysics Toolbox. Behavior Research Methods, Instruments, & Computers, 34(4), 613–617. 10.3758/BF03195489 12564564

[bibr13-1747021820959661] Drieghe D. Rayner K. Pollatsek A. (2005). Eye movements and word skipping during reading revisited. Journal of Experimental Psychology: Human Perception and Performance, 31(5), 954–969. 10.1037/0096-1523.31.5.954 16262491

[bibr14-1747021820959661] Drieghe D. Veldre A. Fitzsimmons G. Ashby J. Andrews S. (2019). The influence of number of syllables on word skipping during reading revisited. Psychonomic Bulletin & Review, 26(2), 616–621. 10.3758/s13423-019-01590-0 30877634

[bibr15-1747021820959661] Engbert R. Nuthmann A. Richter E. M. Kliegl R. (2005). SWIFT: A dynamical model of saccade generation during reading. Psychological Review, 112(4), 777–813. 10.1037/0033-295X.112.4.777 16262468

[bibr16-1747021820959661] Findelsberger E. Hutzler F. Hawelka S. (2019). Spill the load: Mixed evidence for a foveal load effect, reliable evidence for a spillover effect in eye-movement control during reading. Attention, Perception, & Psychophysics, 81(5), 1442–1453. 10.3758/s13414-019-01689-5 PMC6647363 30843176

[bibr17-1747021820959661] Fitzsimmons G. Drieghe D. (2011). The influence of number of syllables on word skipping during reading. Psychonomic Bulletin & Review, 18(4), 736–741. 10.3758/s13423-011-0105-x 21557026

[bibr18-1747021820959661] Gagl B. Hawelka S. Richlan F. Schuster S. Hutzler F. (2014). Parafoveal preprocessing in reading revisited: Evidence from a novel preview manipulation. Journal of Experimental Psychology: Learning, Memory, and Cognition, 40(2), 588–595. 10.1037/a0034408 24041397

[bibr19-1747021820959661] Graupner S. T. Velichkovsky B. M. Pannasch S. Marx J. (2007). Surprise, surprise: Two distinct components in the visually evoked distractor effect. Psychophysiology, 44(2), 251–261. 10.1111/j.1469-8986.2007.00504.x 17343709

[bibr20-1747021820959661] Green P. Macleod C. J. (2016). SIMR: An R package for power analysis of generalized linear mixed models by simulation. Methods in Ecology and Evolution, 7(4), 493–498. 10.1111/2041-210X.12504

[bibr21-1747021820959661] Henderson J. M. Ferreira F. (1990). Effects of foveal processing difficulty on the perceptual span in reading: Implications for attention and eye movement control. Journal of Experimental Psychology: Learning, Memory, and Cognition, 16(3), 417–429. 10.1037/0278-7393.16.3.417 2140401

[bibr22-1747021820959661] Hutzler F. Fuchs I. Gagl B. Schuster S. Richlan F. Braun M. Hawelka S. (2013). Parafoveal X-masks interfere with foveal word recognition: Evidence from fixation-related brain potentials. Frontiers in Systems Neuroscience, 7, e33. 10.3389/fnsys.2013.00033 PMC3719217 23888130

[bibr23-1747021820959661] Hutzler F. Schuster S. Marx C. Hawelka S. (2019). An investigation of parafoveal masks with the incremental boundary paradigm. PLOS ONE, 14(2), e0203013. 10.1371/journal.pone.0203013 PMC6394947 30817789

[bibr24-1747021820959661] Hyönä J. Ekholm M. (2016). Background speech effects on sentence processing during reading: An eye movement study. PLOS ONE, 11(3), e0152133. 10.1371/journal.pone.0152133 PMC4803211 27003410

[bibr25-1747021820959661] Kaakinen J. K. Hyönä J. (2010). Task effects on eye movements during reading. Journal of Experimental Psychology: Learning, Memory, and Cognition, 36(6), 1561–1566. 10.1037/a0020693 20854008

[bibr26-1747021820959661] Kliegl R. Hohenstein S. Yan M. McDonald S. A. (2013). How preview space/time translates into preview cost/benefit for fixation durations during reading. Quarterly Journal of Experimental Psychology, 66(3), 581–600. 10.1080/17470218.2012.658073 22515948

[bibr27-1747021820959661] Leinenger M. (2019). Survival analyses reveal how early phonological processing affects eye movements during reading. Journal of Experimental Psychology: Learning, Memory, and Cognition, 45(7), 1316–1344. 10.1037/xlm0000648 PMC6348047 30047769

[bibr28-1747021820959661] Marois A. Vachon F. (2018). Can pupillometry index auditory attentional capture in contexts of active visual processing? Journal of Cognitive Psychology, 30(4), 484–502. 10.1080/20445911.2018.1470518

[bibr29-1747021820959661] Marx C. Hawelka S. Schuster S. Hutzler F. (2015). An incremental boundary study on parafoveal preprocessing in children reading aloud: Parafoveal masks overestimate the preview benefit. Journal of Cognitive Psychology, 27(5), 549–561. 10.1080/20445911.2015.1008494 26246890 PMC4487581

[bibr30-1747021820959661] Marx C. Hawelka S. Schuster S. Hutzler F. (2017). Foveal processing difficulty does not affect parafoveal preprocessing in young readers. Scientific Reports, 7, e41602. 10.1038/srep41602 PMC5282480 28139718

[bibr31-1747021820959661] Marx C. Hutzler F. Schuster S. Hawelka S. (2016). On the development of parafoveal preprocessing: Evidence from the incremental boundary paradigm. Frontiers in Psychology, 7, e514. 10.3389/fpsyg.2016.00514 PMC4830847 27148123

[bibr32-1747021820959661] MathWorks. (2014). Matlab R2014a [Computer software].

[bibr33-1747021820959661] McDonald S. A. (2006). Parafoveal preview benefit in reading is only obtained from the saccade goal. Vision Research, 46(26), 4416–4424. 10.1016/j.visres.2006.08.027 17045323

[bibr34-1747021820959661] Miellet S. Sparrow L. (2004). Phonological codes are assembled before word fixation: Evidence from boundary paradigm in sentence reading. Brain and Language, 90(1–3), 299–310. 10.1016/S0093-934X(03)00442-5 15172547

[bibr35-1747021820959661] Pelli D. G. (1997). The VideoToolbox software for visual psychophysics: Transforming numbers into movies. Spatial Vision, 10(4), 437–442. 10.1163/156856897X00366 9176953

[bibr36-1747021820959661] Pereira E. J. Castelhano M. S. (2019). Attentional capture is contingent on scene region: Using surface guidance framework to explore attentional mechanisms during search. Psychonomic Bulletin & Review, 26, 1273–1281. 10.3758/s13423-019-01610-z 31161527

[bibr37-1747021820959661] Pollatsek A. Lesch M. Morris R. K. Rayner K. (1992). Phonological codes are used in integrating information across saccades in word identification and reading. Journal of Experimental Psychology: Human Perception and Performance, 18(1), 148–162. 10.1037/0096-1523.18.1.148 1532185

[bibr38-1747021820959661] Pollatsek A. Reichle E. D. Rayner K. (2006). Tests of the E-Z Reader model: Exploring the interface between cognition and eye-movement control. Cognitive Psychology, 52(1), 1–56. 10.1016/j.cogpsych.2005.06.001 16289074

[bibr39-1747021820959661] R Core Team. (2018). R: A language and environment for statistical computing. R Foundation for Statistical Computing. http://www.r-project.org/

[bibr40-1747021820959661] Radach R. Huestegge L. Reilly R. (2008). The role of global top-down factors in local eye-movement control in reading. Psychological Research, 72(6), 675–688. 10.1007/s00426-008-0173-3 18936964

[bibr41-1747021820959661] Rastle K. Harrington J. Coltheart M. (2002). 358,534 nonwords: The ARC nonword database. Quarterly Journal of Experimental Psychology, 55(4), 1339–1362. 10.1080/02724980244000099 12420998

[bibr42-1747021820959661] Rayner K. (1975). The perceptual span and peripheral cues in reading. Cognitive Psychology, 81(7), 65–81. 10.1016/0010-0285(75)90005-5

[bibr43-1747021820959661] Rayner K. (1998). Eye movements in reading and information processing: 20 years of research. Psychological Bulletin, 124(3), 372–422. 10.1037/0033-2909.124.3.372 9849112

[bibr44-1747021820959661] Rayner K. (2009). Eye movements and attention in reading, scene perception, and visual search. Quarterly Journal of Experimental Psychology, 62(8), 1457–1506. 10.1080/17470210902816461 19449261

[bibr45-1747021820959661] Rayner K. McConkie G. W. Zola D. (1980). Integrating information across eye movements. Cognitive Psychology, 12(2), 206–226. 10.1016/0010-0285(80)90009-2 7371377

[bibr46-1747021820959661] Rayner K. White S. J. Kambe G. Miller B. Liversedge S. P. (2003). On the processing of meaning from parafoveal vision during eye fixations in reading. In Hyönä J. Radach R. Deubel H. (Eds.), The mind’s eye: Cognitive and applied aspects of eye movement research (pp. 213–234). Elsevier. 10.1016/B978-044451020-4/50013-X

[bibr47-1747021820959661] Reichle E. D. Pollatsek A. Fisher D. L. Rayner K. (1998). Toward a model of eye movement control in reading. Psychological Review, 105(1), 125–157. 10.1037/0033-295X.105.1.125 9450374

[bibr48-1747021820959661] Reichle E. D. Reingold E. M. (2013). Neurophysiological constraints on the eye-mind link. Frontiers in Human Neuroscience, 7, e361. 10.3389/fnhum.2013.00361 PMC3710954 23874281

[bibr49-1747021820959661] Risse S. Hohenstein S. Kliegl R. Engbert R. (2014). A theoretical analysis of the perceptual span based on SWIFT simulations of the n + 2 boundary paradigm. Visual Cognition, 22(3–4), 283–308. 10.1080/13506285.2014.881444 24771996 PMC3996545

[bibr50-1747021820959661] Schotter E. R. Angele B. Rayner K. (2012). Parafoveal processing in reading. Attention, Perception & Psychophysics, 74(1), 5–35. 10.3758/s13414-011-0219-2 22042596

[bibr51-1747021820959661] Schotter E. R. Bicknell K. Howard I. Levy R. Rayner K. (2014). Task effects reveal cognitive flexibility responding to frequency and predictability: Evidence from eye movements in reading and proofreading. Cognition, 131(1), 1–27. 10.1016/j.cognition.2013.11.018 24434024 PMC3943895

[bibr52-1747021820959661] Schotter E. R. Reichle E. D. Rayner K. (2014). Rethinking parafoveal processing in reading: Serial-attention models can explain semantic preview benefit and N +2 preview effects. Visual Cognition, 22(3–4), 309–333. 10.1080/13506285.2013.873508

[bibr53-1747021820959661] Sheridan H. Reichle E. D. (2016). An analysis of the time course of lexical processing during reading. Cognitive Science, 40(3), 522–553. 10.1111/cogs.12246 25939443 PMC5122144

[bibr54-1747021820959661] Slattery T. J. Angele B. Rayner K. (2011). Eye movements and display change detection during reading. Journal of Experimental Psychology: Human Perception and Performance, 37(6), 1924–1938. 10.1037/a0024322 21688934

[bibr55-1747021820959661] Slattery T. J. Schotter E. R. Berry R. W. Rayner K. (2011). Parafoveal and foveal processing of abbreviations during eye fixations in reading: Making a case for case. Journal of Experimental Psychology: Learning, Memory, and Cognition, 37(4), 1022–1031. 10.1037/a0023215 PMC3130820 21480754

[bibr56-1747021820959661] Snell J. van Leipsig S. Grainger J. Meeter M. (2018). OB1-reader: A model of word recognition and eye movements in text reading. Psychological Review, 125(6), 969–984. 10.1037/rev0000119 30080066

[bibr57-1747021820959661] Sokolov E. N. (1963). Higher nervous functions: The orienting reflex. Annual Review of Physiology, 25(1), 545–580. 10.1146/annurev.ph.25.030163.002553 13977960

[bibr58-1747021820959661] Sokolov E. N. (2001). Orienting response. In Smelser N. J. Baltes P. B. (Eds.), International encyclopedia of the social & behavioral sciences (pp. 10978–10981). Elsevier Science. 10.1016/B0-08-043076-7/03536-1

[bibr59-1747021820959661] Stracuzzi D. J. (2004). EyeTrack (Version 0.7.10h) [Computer software]. http://blogs.umass.edu/eyelab

[bibr60-1747021820959661] Theeuwes J. Kramer A. F. Hahn S. Irwin D. E. (1998). Our eyes do not always go where we want them to go: Capture of the eyes by new objects. Psychological Science, 9(5), 379–385. 10.1111/1467-9280.00071

[bibr61-1747021820959661] Vasilev M. R. Angele B. (2017). Parafoveal preview effects from word N + 1 and word N + 2 during reading: A critical review and Bayesian meta-analysis. Psychonomic Bulletin & Review, 24(3), 666–689. 10.3758/s13423-016-1147-x 27576520

[bibr62-1747021820959661] Vasilev M. R. Liversedge S. P. Rowan D. Kirkby J. A. Angele B. (2019). Reading is disrupted by intelligible background speech: Evidence from eye-tracking. Journal of Experimental Psychology: Human Perception and Performance, 45, 1484–1512. 10.1037/xhp0000680 31436455

[bibr63-1747021820959661] Vasilev M. R. Parmentier F. B. Angele B. Kirkby J. A. (2019). Distraction by deviant sounds during reading: An eye-movement study. Quarterly Journal of Experimental Psychology, 72(7), 1863–1875. 10.1177/1747021818820816 PMC6613176 30518304

[bibr64-1747021820959661] Vasilev M. R. Slattery T. J. Kirkby J. A. Angele B. (2018). What are the costs of degraded parafoveal previews during silent reading? Journal of Experimental Psychology: Learning, Memory, and Cognition, 44(3), 371–386. 10.1037/xlm0000433 28661179

[bibr65-1747021820959661] Vasilev M. R. Yates M. Slattery T. J. (2019). Do readers integrate phonological codes across saccades? A Bayesian meta-analysis and a survey of the unpublished literature. Journal of Cognition, 2(1), 1–29. 10.5334/joc.87 31750415 PMC6838770

[bibr66-1747021820959661] Veldre A. Andrews S. (2018). How does foveal processing difficulty affect parafoveal processing during reading? Journal of Memory and Language, 103, 74–90. 10.1016/j.jml.2018.08.001

[bibr67-1747021820959661] von der Malsburg T. Angele B. (2017). False positives and other statistical errors in standard analyses of eye movements in reading. Journal of Memory and Language, 94, 119–133. 10.1016/j.jml.2016.10.003 28603341 PMC5461930

[bibr68-1747021820959661] Warrington K. L. McGowan V. A. Paterson K. B. White S. J. (2018). Effects of aging, word frequency, and text stimulus quality on reading across the adult lifespan: Evidence from eye movements. Journal of Experimental Psychology: Learning, Memory, and Cognition, 44(11), 1714–1729. 10.1037/xlm0000543 PMC6233613 29672115

[bibr69-1747021820959661] Weiss A. F. Kretzschmar F. Schlesewsky M. Bornkessel-Schlesewsky I. D. Staub A. (2018). Comprehension demands modulate re-reading, but not first pass reading behavior. Quarterly Journal of Experimental Psychology, 71, 198–210. 10.1080/17470218.2017.1307862 28300468

[bibr70-1747021820959661] White S. J. Rayner K. Liversedge S. P. (2005). Eye movements and the modulation of parafoveal processing by foveal processing difficulty: A reexamination. Psychonomic Bulletin & Review, 12(5), 891–896. 10.3758/BF03196782 16524007

[bibr71-1747021820959661] Wotschack C. Kliegl R. (2013). Reading strategy modulates parafoveal-on-foveal effects in sentence reading. Quarterly Journal of Experimental Psychology, 66(3), 548–562. 10.1080/17470218.2011.625094 22026498

[bibr72-1747021820959661] Yan G. Meng Z. Liu N. He L. Paterson K. B. (2018). Effects of irrelevant background speech on eye movements during reading. Quarterly Journal of Experimental Psychology, 71(6), 1270–1275. 10.1080/17470218.2017.1339718 28590881

[bibr73-1747021820959661] Yantis S. Jonides J. (1984). Abrupt visual onsets and selective attention: Evidence from visual search. Journal of Experimental Psychology: Human Perception and Performance, 10(5), 601–621. 10.1037/0096-1523.10.5.601 6238122

[bibr74-1747021820959661] Zhang H. Miller K. Cleveland R. Cortina K. (2018). How listening to music affects reading: Evidence from eye tracking. Journal of Experimental Psychology: Learning, Memory, and Cognition, 44(11), 1778–1791. 10.1037/xlm0000544 29389184

