# Supplementary material for: Parafoveal degradation during reading reduces preview costs only when it is not perceptually distinct
Source: Q J Exp Psychol (Hove). 2020 Sep 28;74(2):254–76. doi: 10.1177/1747021820959661 (PMC8044602; doi:10.1177/1747021820959661)
Supplement: QJE-STD-19-283.R2-Supplementary_Material – Supplemental material for Parafoveal degradation during reading reduces preview costs only when it is not perceptually distinct [file QJE-STD-19-283.R2-Supplementary_Material.docx]

Supplementary Material for:

**Parafoveal degradation during reading reduces preview costs only when it is not perceptually distinct**

Martin R. Vasilev, Mark Yates, Ethan Prueitt, and Timothy J. Slattery

**Supplementary Material 1**

**Comparison between Experiment 1a and Experiment 1b**

Table A1

*LMM Result for the Target Word Comparison between Experiment 1a (All Words Degraded) and Experiment 1b (Target Word and Rest of Sentence Degraded)*

| Fixed effects | FFD | | | |  | SFD | | | |  | GD | | | |
| --- | --- | --- | --- | --- | --- | --- | --- | --- | --- | --- | --- | --- | --- | --- |
|  | b | SE | | t |  | b | | SE | t |  | b | | SE | t |
| Intercept | 5.45 | .011 | | **481.7** |  | 5.49 | | .013 | **420.3** |  | 5.55 | | .014 | **387.5** |
| Invalid Prev. | .064 | .01 | | **6.161** |  | .094 | | .011 | **8.306** |  | .109 | | .012 | **9.451** |
| Orth Prev. | .03 | .01 | | **2.863** |  | .047 | | .011 | **4.131** |  | .058 | | .012 | **5.025** |
| Phon Prev. | -.005 | .01 | | -.463 |  | .002 | | .011 | .184 |  | .002 | | .012 | .2 |
| Deg | -.019 | .004 | | **-4.49** |  | -.015 | | .004 | **-3.37** |  | -.013 | | .005 | **-2.71** |
| Exp | .026 | .004 | | **6.88** |  | .029 | | .004 | **7.129** |  | .031 | | .004 | **7.485** |
| Invalid Prev. x Deg | .019 | .01 | | 1.845 |  | .031 | | .011 | **2.761** |  | .036 | | .012 | **3.076** |
| Orth Prev. x Deg | .007 | .01 | | .639 |  | .012 | | .011 | 1.06 |  | .009 | | .012 | .797 |
| Phon. Prev. x Deg | .005 | .01 | | .475 |  | .009 | | .011 | .797 |  | .019 | | .012 | 1.597 |
| Invalid Prev. x Exp | .004 | .01 | | .409 |  | .007 | | .011 | .663 |  | -.005 | | .012 | -.458 |
| Orth Prev. x Exp | .003 | .01 | | .303 |  | .001 | | .011 | .058 |  | -.009 | | .012 | -.749 |
| Phon Prev. x Exp | .007 | .01 | | .63 |  | .002 | | .011 | .191 |  | .001 | | .012 | .052 |
| Deg x Exp | -.008 | .004 | | **-2.17** |  | -.013 | | .004 | **-3.16** |  | -.011 | | .004 | **-2.66** |
| Invalid Prev. x Deg x Exp | -.02 | .01 | | -1.93 |  | -.019 | | .011 | -1.691 |  | -.002 | | .012 | -.175 |
| Orth Prev. x Deg x Exp | -.019 | .01 | | -1.798 |  | -.017 | | .011 | -1.454 |  | .001 | | .012 | .061 |
| Phon Prev. x Deg x Exp | .015 | .01 | | 1.471 |  | .008 | | .011 | .687 |  | -.0003 | | .012 | -.029 |
| Random effects | Var. | | SD | Corr. |  | Var. | SD | | Corr. |  | Var. | SD | | Corr. |
| Intercept (items) | .0038 | | .0616 |  |  | .0056 | .0749 | |  |  | .0076 | .0872 | |  |
| Deg (items) | .0001 | | .0109 | .52 |  |  |  | |  |  | .0002 | .0153 | | .52 |
| Intercept (subj) | .0043 | | .0654 |  |  | .0054 | .0733 | |  |  | .0059 | .0771 | |  |
| Deg (subj) | .0001 | | .0120 | -0.13 |  | .0002 | .0133 | | .13 |  | .0003 | .0163 | | -.04 |
| Residual | .0906 | | .3010 |  |  | .0830 | .2880 | |  |  | .1135 | .3369 | |  |

*Note*: Invalid Prev.: Invalid preview effect (letter mask vs valid preview). Orth Prev: orthographic preview effect (orthographic vs letter mask preview). Phon Prev: phonological preview effect (phonological vs orthographic preview). Deg: preview degradation. Exp: Experiment. FFD: first fixation duration. SFD: single fixation duration. GD: gaze duration. subj: subjects. Statistically significant *t*-values are formatted in bold.

To compare how the results differed between Experiment 1a and 1b, the two datasets were merged and experiment number was added as a fixed effect in the models (contrast coding: Experiment 1a: -1; Experiment 1b: 1). The results are shown in Table A1. Consistent with the main results, there were main effects of preview effect and orthographic preview across the two experiments. The main effect of degradation was also significant, which indicates longer fixation durations following degraded compared to non-degraded previews. Additionally, there was a main effect of Experiment, which was due to longer fixation durations in Experiment 1b compared to Experiment 1a. Importantly, there was an interaction between degradation and Experiment, which was due to longer fixation durations after degraded previews in Experiment 1b compared to Experiment 1a. Therefore, this suggests that degradation influenced fixation durations to a greater extent in Experiment 1b (where target word degradation was perceptually distinct) compared to Experiment 1a (where all words in the sentence were degraded and thus target word degradation was “hidden”).

**Comparison between Experiment 2a and Experiment 2b**

The comparison of the two replication experiments (2a and 2b) is presented in Table A2. Similar to the results from the individual studies, the invalid preview and orthographic preview effects were significant, and so was their interaction with degradation. Consistent with Experiment 1, the main effect of degradation was also significant, which was due to longer fixation durations in the degraded compared to the non-degraded condition. This was again driven by the increase in fixation durations with degradation in Experiment 2b, as there was no main effect of degradation in the individual-study analysis of Experiment 2a (see Table 4). Additionally, the main effect Experiment was again significant, which was due to longer fixation durations in Experiment 2b compared to Experiment 2a. Critically, however, the interaction between degradation and Experiment was again significant. This occurred because degradation led to a greater increase in fixation durations when target word degradation was distinct (Experiment 2b) compared to when it was “hidden” by degrading all words in the sentence (Experiment 2a). In summary, the results from four experiments suggest that parafoveal degradation leads to inflated fixation durations only when the target word manipulation is perceptually distinct.

Table A2

*LMM Result for the Target Word Comparison between Experiment 2a (All Words Degraded) and Experiment 2b (Target Word and Rest of Sentence Degraded)*

| Fixed effects | FFD | | | |  | SFD | | | |  | GD | | | |
| --- | --- | --- | --- | --- | --- | --- | --- | --- | --- | --- | --- | --- | --- | --- |
|  | b | SE | | t |  | b | | SE | t |  | b | | SE | t |
| Intercept | 5.53 | .01 | | **457.5** |  | 5.55 | | .01 | **440.1** |  | 5.59 | | .01 | **431.1** |
| Invalid Prev. | .10 | .01 | | **12.9** |  | .12 | | .01 | **14.9** |  | .12 | | .01 | **14.82** |
| Orth Prev. | .05 | .01 | | **6.3** |  | .06 | | .01 | **7.59** |  | .07 | | .01 | **8.37** |
| Deg | -.02 | < .01 | | **-7.17** |  | -.02 | | < .01 | **-6.87** |  | -.02 | | < .01 | **-5.79** |
| Exp | .02 | < .01 | | **5.91** |  | .02 | | < .01 | **5.93** |  | .02 | | < .01 | **6.58** |
| Invalid Prev. x Deg | .04 | .01 | | **5.84** |  | .05 | | .01 | **6.49** |  | .05 | | .01 | **6.68** |
| Orth Prev. x Deg | .03 | .01 | | **3.38** |  | .03 | | .01 | **3.6** |  | .03 | | .01 | **3.73** |
| Invalid Prev. x Exp | < .01 | .01 | | .38 |  | < .01 | | .01 | .25 |  | < -.01 | | .01 | -.06 |
| Orth Prev. x Exp | .01 | .01 | | 1.76 |  | .01 | | .01 | 1.27 |  | .01 | | .01 | .79 |
| Deg x Exp | -.02 | < .01 | | **-6.62** |  | -.02 | | < .01 | **-6.44** |  | -.02 | | < .01 | **-6.98** |
| Invalid Prev. x Deg x Exp | -.01 | .01 | | -1.17 |  | -.01 | | .01 | -1.45 |  | -.01 | | .01 | -.67 |
| Orth Prev. x Deg x Exp | < -.01 | .01 | | -.24 |  | < -.01 | | .01 | -.56 |  | -.01 | | .01 | -.89 |
| Random effects | Var. | | SD | Corr. |  | Var. | SD | | Corr. |  | Var. | SD | | Corr. |
| Intercept (items) | .0041 | | .0641 |  |  | .0048 | .0692 | |  |  | .0065 | .0806 | |  |
| Deg (items) | .00001 | | .0039 | -.14 |  |  |  | |  |  |  |  | |  |
| Intercept (subj) | .0064 | | .0797 |  |  | .0068 | .0825 | |  |  | .0066 | .0811 | |  |
| Deg (subj) |  | |  |  |  | .0001 | .0108 | |  |  | 0.0001 | .0095 | | -.49 |
| Residual | .0978 | | .3128 |  |  | .0939 | .3065 | |  |  | .1086 | .3296 | |  |

*Note*: Invalid Prev.: Invalid preview effect (letter mask vs valid preview). Orth Prev: orthographic preview effect (orthographic vs letter mask preview). Deg: preview degradation. Exp: Experiment. FFD: first fixation duration. SFD: single fixation duration. GD: gaze duration. subj: subjects. Statistically significant *t*-values are formatted in bold.

**Supplementary Material 2**

**Statistical Power Simulations for Experiment 2**

Statistical power for Experiments 2a and 2b was calculated based on the data from Experiment 1a and 1b, respectively. The power calculations were done with LMMs using the *simr* R package v.1.0.5 (Green & Macleod, 2016). This package calculates empirical power by running a large number of simulated models and calculating what percentage of them returned a significant effect (e.g., if 50 out of 100 models yield a significant result, the power would be 0.5). The current calculations are based on 500 simulated models with 60 subjects in both experiments. While Experiments 2a and 2b had 23 items per condition, the simulations were based only on 17 items per condition in order to account for an expected 26% data loss during the pre-processing stages.

Table S1

*Mean Statistical Power for Experiment 2a (a Replication of Experiment 1a), Estimated with 60 Subjects and 17 Items per Condition*

| Effect | FFD | | SFD | | GD | |
| --- | --- | --- | --- | --- | --- | --- |
|  | Mean | 95 % CI | Mean | 95 % CI | Mean | 95 % CI |
| **Invalid Prev** | **>0.999** | **[0.992, 1]** | **>0.999** | **[0.992, 1]** | **>0.999** | **[0.992, 1]** |
| **Orth Prev** | **0.898** | **[0.868, 0.923]** | **>0.999** | **[0.992, 1]** | **>0.999** | **[0.992]** |
| Deg | 0.96 | [0.939, 0.975] | 0.132 | [0.104 0.165] | 0.052 | [0.034 0.075] |
| **Invalid Prev x Deg** | **0.98** | **[0.974, 0.996]** | **>0.999** | **[0.992, 1]** | **0.972** | **[0.953 0.985]** |
| **Orth Prev x Deg** | **0.822** | **[0.786, 0.855]** | **0.938** | **[0.913, 0.957]** | 0.166 | [0.134 0.202] |

*Note*: The power for all effects is provided for completeness. However, only the effects formatted in the bold were predicted in the experiment. An Orth Prev x Deg interaction in GD was originally predicted for Experiment 1, but the effect was not found in the data. The power simulations further suggested that the effect is unlikely to exist. Seventeen items were used to represent an expected data loss of 26%.

The power results are reported in Tables S1 for Experiment 2a and Table S2 for Experiment 2b. The effects of interest are formatted in bold. Experiment 2a had an average power of 0.964 (*SD*= 0.057; range 0.822- 0.999) and Experiment 2b had an average power of 0.991 (*SD*= 0.022; range= 0.932-0.999) to find the predicted effects with 60 subjects. Therefore, a sample size of 60 was chosen because it had a very high power of detecting the predicted effects.

Table S2

*Mean Statistical Power for Experiment 2b (a Replication of Experiment 1b), Estimated with 60 Subjects and 17 Items per Condition*

| Effect | FFD | | SFD | | GD | |
| --- | --- | --- | --- | --- | --- | --- |
|  | Mean | 95 % CI | Mean | 95 % CI | Mean | 95 % CI |
| **Invalid Prev** | **>0.999** | **[0.992, 1]** | **>0.999** | **[0.992, 1]** | **>0.999** | **[0.992, 1]** |
| **Orth Prev** | **0.932** | **[0.906, 0.952]** | **0.998** | **[0.989, >0.999]** | **>0.999** | **[0.992, 1]** |
| **Deg** | **>0.999** | **[0.992, 1]** | **>0.999** | **[0.992, 1]** | **0.998** | **[0.989, >0.999]** |
| Invalid Prev x Deg | 0.1 | [0.075, 0.129] | 0.672 | [0.628, 0.713] | 0.93 | [0.904, 0.951] |
| Orth Prev x Deg | 0.142 | [0.113, 0.176] | 0.044 | [0.028, 0.066] | 0.238 | [0.201, 0.278] |

*Note*: The power for all effects is provided for completeness. However, only the effects formatted in the bold were predicted in the experiment. Seventeen items were used to represent an expected data loss of 26%.

**Parafoveal-on-Foveal Effects on the Pre-target Word (Post-hoc)**

Post-hoc analyses were carried out to investigate potential influences of target word preview on pre-target word fixation durations (i.e., *parafoveal-on-foveal [PoF] effects*; see Drieghe, 2012 and Brothers, Hoversten, & Traxler, 2017 for an overview). To our knowledge, no studies using Marx et al.'s (2015) incremental boundary paradigm have shown that visual degradation can cause PoF effects. However, Hutzler, Schuster, Marx, & Hawelka (2019, Experiment 4) found that degradation *reduced* the letter mask PoF effect found on the pre-target word. As such, similar analyses could also be useful in the present experiments.

Mean fixation durations on the pre-target word for the four experiments are reported in Table S3. The LMM results are shown in Tables S4-S7. In Experiment 1a, where all words were degraded prior to their fixation, the only significant PoF effect was that of phonological preview on SFD. This was due to longer SFD on the pre-target word in the phonological compared to the orthographic preview condition. In Experiment 2a, which was a high-power replication of Experiment 1a, there were no significant PoF effects (note that this experiment did not have a phonological preview condition).

Experiment 1b, in which only the target word and remaining sentence were degraded, also showed no significant PoF effects. In Experiment 2b, which was a high-power replication of Experiment 1b, there was a significant orthographic preview PoF effect in SFD and GD. This was due to longer fixation durations in the letter mask preview condition compared to the orthographic preview condition. However, the invalid preview PoF effect (letter mask vs valid preview) was not statistically significant. There were no other PoF effects.

Table S3

*Mean Fixation Durations on the Pre-target Word in Experiments 1-2 (Standard Deviations in Parenthesis)*

| Degradation | Preview type | FFD | SFD | GD |
| --- | --- | --- | --- | --- |
| **Experiment 1a** | | | | |
| 0% | valid | 217 (72) | 218 (72) | 241 (103) |
| 0% | phonological | 221 (74) | 222 (76) | 247 (104) |
| 0% | orthographic | 214 (64) | 212 (63) | 248 (121) |
| 0% | letter mask | 217 (67) | 218 (65) | 247 (113) |
| 20% | valid | 212 (58) | 214 (58) | 235 (86) |
| 20% | phonological | 221 (67) | 223 (68) | 245 (95) |
| 20% | orthographic | 221 (72) | 221 (72) | 242 (108) |
| 20% | letter mask | 217 (64) | 218 (62) | 239 (100) |
| **Experiment 1b** | | | | |
| 0% | valid | 225 (79) | 226 (78) | 252 (118) |
| 0% | phonological | 221 (73) | 222 (68) | 244 (101) |
| 0% | orthographic | 226 (76) | 225 (76) | 247 (108) |
| 0% | letter mask | 219 (68) | 219 (63) | 248 (104) |
| 20% | valid | 217 (64) | 217 (61) | 241 (112) |
| 20% | phonological | 228 (82) | 226 (83) | 246 (104) |
| 20% | orthographic | 222 (70) | 222 (70) | 249 (111) |
| 20% | letter mask | 226 (72) | 228 (74) | 252 (117) |
| **Experiment 2a (replication of 1a)** | | | | |
| 0% | valid | 228 (75) | 229 (74) | 248 (97) |
| 0% | orthographic | 227 (76) | 229 (77) | 244 (97) |
| 0% | letter mask | 230 (86) | 231 (86) | 248 (110) |
| 20% | valid | 231 (76) | 232 (77) | 250 (99) |
| 20% | orthographic | 229 (68) | 230 (68) | 248 (94) |
| 20% | letter mask | 228 (70) | 230 (71) | 246 (94) |
| **Experiment 2b (replication of 1b)** | | | | |
| 0% | valid | 234 (74) | 233 (73) | 257 (115) |
| 0% | orthographic | 234 (83) | 233 (82) | 255 (117) |
| 0% | letter mask | 240 (83) | 241 (85) | 268 (122) |
| 20% | valid | 236 (83) | 237 (84) | 255 (109) |
| 20% | orthographic | 230 (75) | 231 (76) | 252 (104) |
| 20% | letter mask | 235 (85) | 236 (85) | 256 (107) |

To summarise, there was little evidence for PoF effects in the present research. There were only two PoF effects: one caused by the phonological preview condition in Experiment 1a and another one caused by the letter mask condition in Experiment 2b. These effects may have originated if readers perceived unusual letter combinations in the parafovea while fixating the pre-target word. Such orthographic PoF effects are not uncommon and have sporadically been reported in the literature (e.g., Angele, Slattery, & Rayner, 2016; Hutzler et al., 2019, Experiment 4; Inhoff, Starr, & Shindler, 2000; Starr & Inhoff, 2004; Veldre & Andrews, 2018, Experiment 2). Critically, however, degradation did not cause PoF effects in any of the experiments. This is consistent with Vasilev, Slattery, Kirkby, and Angele' s (2018) results. This suggests that parafoveal degradation in the incremental boundary paradigm (Marx et al., 2015) does not affect reading behaviour on the pre-target word.

Table S4

*LMM Results for Fixation Durations on the Pre-Target Word in Experiment 1a*

| Fixed effects | FFD | | | |  | SFD | | | |  | GD | | | |
| --- | --- | --- | --- | --- | --- | --- | --- | --- | --- | --- | --- | --- | --- | --- |
|  | b | SE | | t |  | b | | SE | t |  | b | SE | | t |
| Intercept | 5.35 | .01 | | **371.2** |  | 5.36 | | .02 | **348.2** |  | 5.43 | .02 | | **293.7** |
| Invalid Prev. | .01 | .01 | | .87 |  | .01 | | .01 | .42 |  | .01 | .02 | | .84 |
| Orth Prev. | < .01 | .01 | | .14 |  | .01 | | .01 | .61 |  | < .01 | .02 | | .23 |
| Phon Prev. | -0.02 | .01 | | -1.3 |  | -.03 | | .01 | **-2** |  | -.02 | .02 | | -1.29 |
| Deg | < -.01 | .01 | | -.88 |  | < -.01 | | .01 | -.6 |  | .01 | .01 | | 1.03 |
| Invalid Prev. x Deg | < -.01 | .01 | | -.26 |  | -.01 | | .01 | -.41 |  | < .01 | .02 | | .27 |
| Orth Prev. x Deg | .01 | .01 | | .93 |  | .01 | | .01 | .96 |  | < .01 | .02 | | .23 |
| Phon Prev. x Deg | -.01 | .01 | | -.91 |  | -.01 | | .01 | -.88 |  | .01 | .02 | | .41 |
| Random effects | Var. | | SD | Corr. |  | Var. | SD | | Corr. |  | Var. | | SD | Corr. |
| Intercept (items) | .0036 | | .0598 |  |  | .0048 | .0690 | |  |  | .0085 | | .0921 |  |
| Deg (items) | .0002 | | .0126 | 1 |  | .0004 | .0194 | | .79 |  |  | |  |  |
| Intercept (subj) | .0090 | | .0951 |  |  | .0097 | .0988 | |  |  | .0129 | | .1134 |  |
| Deg (subj) | .0006 | | .0248 |  |  | .0007 | .0267 | | -.29 |  | .0006 | | .0245 | .04 |
| Residual | .0637 | | .2524 |  |  | .0592 | .2434 | |  |  | .1032 | | .3213 |  |

*Note*: Invalid Prev.: Invalid preview effect (letter mask vs valid preview). Orth Prev: orthographic preview effect (orthographic vs letter mask preview). Phon Prev.: phonological preview effect (phonological vs. orthographic preview). Deg: preview degradation. FFD: first fixation duration. SFD: single fixation duration. GD: gaze duration. subj: subjects. Statistically significant *t*-values are formatted in bold.

Table S5

*LMM Results for Fixation Durations on the Pre-Target Word in Experiment 1b*

| Fixed effects | FFD | | | |  | SFD | | | |  | GD | | | |
| --- | --- | --- | --- | --- | --- | --- | --- | --- | --- | --- | --- | --- | --- | --- |
|  | b | SE | | t |  | b | | SE | t |  | b | SE | | t |
| Intercept | 5.36 | .02 | | **321.3** |  | 5.37 | | .02 | **305.6** |  | 5.43 | .02 | | **276.4** |
| Invalid Prev. | < .01 | .01 | | .38 |  | .01 | | .01 | .44 |  | .01 | .02 | | .77 |
| Orth Prev. | < -.01 | .01 | | -.3 |  | < -.01 | | .01 | -.1 |  | < .01 | .02 | | .25 |
| Phon Prev. | < -.01 | .01 | | -.17 |  | < -.01 | | .01 | -.08 |  | .01 | .02 | | .42 |
| Deg | < -.01 | .01 | | -.37 |  | < -.01 | | < .01 | -.21 |  | < .01 | .01 | | .38 |
| Invalid Prev. x Deg | -.02 | .01 | | -1.23 |  | -.02 | | .01 | -1.68 |  | -.01 | .02 | | -.69 |
| Orth Prev. x Deg | -.02 | .01 | | -1.56 |  | -.02 | | .01 | -1.19 |  | -.01 | .02 | | -.35 |
| Phon Prev. x Deg | .02 | .01 | | 1.61 |  | .01 | | .01 | .64 |  | .01 | .02 | | .35 |
| Random effects | Var. | | SD | Corr. |  | Var. | SD | | Corr. |  | Var. | | SD | Corr. |
| Intercept (items) | .0046 | | .0676 |  |  | .0047 | .0684 | |  |  | .0088 | | .0937 |  |
| Deg (items) |  | |  |  |  |  |  | |  |  |  | |  |  |
| Intercept (subj) | .0127 | | .1126 |  |  | .0143 | .1197 | |  |  | .0153 | | .1239 |  |
| Deg (subj) | .0002 | | .0158 | .18 |  |  |  | |  |  | .0005 | | .0216 | .06 |
| Residual | .0667 | | .2583 |  |  | .0617 | .2484 | |  |  | .1058 | | .3252 |  |

*Note*: Invalid Prev.: Invalid preview effect (letter mask vs valid preview). Orth Prev: orthographic preview effect (orthographic vs letter mask preview). Phon Prev.: phonological preview effect (phonological vs. orthographic preview). Deg: preview degradation. FFD: first fixation duration. SFD: single fixation duration. GD: gaze duration. subj: subjects. Statistically significant *t*-values are formatted in bold.

Table S6

*LMM Results for Fixation Durations on the Pre-Target Word in Experiment 2a*

| Fixed effects | FFD | | | |  | SFD | | | |  | GD | | | |
| --- | --- | --- | --- | --- | --- | --- | --- | --- | --- | --- | --- | --- | --- | --- |
|  | b | SE | | t |  | b | | SE | t |  | b | SE | | t |
| Intercept | 5.39 | .02 | | **346.8** |  | 5.4 | | .02 | **339.4** |  | 5.45 | .02 | | **308.2** |
| Invalid Prev. | < -.01 | .01 | | -.15 |  | < .01 | | .01 | .04 |  | -.01 | .01 | | -.88 |
| Orth Prev. | < .01 | .01 | | .29 |  | < .01 | | .01 | .43 |  | < .01 | .01 | | .07 |
| Deg | -.01 | < .01 | | -1.29 |  | -.01 | | < .01 | -1.43 |  | -.01 | .01 | | -1.34 |
| Invalid Prev. x Deg | .01 | .01 | | .67 |  | < .01 | | .01 | .44 |  | < .01 | .01 | | .25 |
| Orth Prev. x Deg | .01 | .01 | | 1.09 |  | .01 | | .01 | .59 |  | .01 | .01 | | 1.04 |
| Random effects | Var. | | SD | Corr. |  | Var. | SD | | Corr. |  | Var. | | SD | Corr. |
| Intercept (items) | .0034 | | .0583 |  |  | .0039 | .0624 | |  |  | .0067 | | .0818 |  |
| Deg (items) | .0007 | | .0272 | .10 |  | .0007 | .0272 | | .09 |  | .0008 | | .0282 | -.05 |
| Intercept (subj) | .0122 | | .1105 |  |  | .0126 | .1125 | |  |  | .0148 | | .1216 |  |
| Deg (subj) | .0002 | | .0161 | .48 |  | .0003 | .0179 | | .52 |  | .0003 | | .0170 | .28 |
| Residual | .0675 | | .2598 |  |  | .0654 | .2556 | |  |  | .0903 | | .3005 |  |

*Note*: Invalid Prev.: Invalid preview effect (letter mask vs valid preview). Orth Prev: orthographic preview effect (orthographic vs letter mask preview). Deg: preview degradation. FFD: first fixation duration. SFD: single fixation duration. GD: gaze duration. subj: subjects. Statistically significant *t*-values are formatted in bold.

Table S7

*LMM Results for Fixation Durations on the Pre-Target Word in Experiment 2b*

| Fixed effects | FFD | | | |  | SFD | | |  | GD | | | |
| --- | --- | --- | --- | --- | --- | --- | --- | --- | --- | --- | --- | --- | --- |
|  | b | SE | t | |  | b | SE | t |  | b | SE | | t |
| Intercept | 5.41 | .02 | **334.3** | |  | 5.42 | .02 | **322.7** |  | 5.48 | .02 | | **287.3** |
| Invalid Prev. | < .01 | .01 | .24 | |  | .01 | .01 | .72 |  | .01 | .01 | | 1.24 |
| Orth Prev. | .02 | .01 | 1.46 | |  | .02 | .01 | **2.04** |  | .03 | .01 | | **2.22** |
| Deg | .01 | < .01 | 1.38 | |  | < .01 | < .01 | .78 |  | .01 | .01 | | 1.56 |
| Invalid Prev. x Deg | .02 | .01 | 1.86 | |  | .02 | .01 | 1.88 |  | .02 | .01 | | 1.88 |
| Orth Prev. x Deg | < .01 | .01 | .48 | |  | .01 | .01 | .77 |  | .01 | .01 | | 1.27 |
| Random effects | Var. | SD | Corr. | |  | Var. | SD | Corr. |  | Var. | | SD | Corr. |
| Intercept (items) | .0053 | .0731 |  | |  | .0061 | .0779 |  |  | .0109 | | .1045 |  |
| Deg (items) | .0001 | .0116 | .08 | |  |  |  |  |  | .0002 | | .0151 | .56 |
| Invalid Prev. (items) | .0008 | .0289 | .73 | .48 |  |  |  |  |  |  | |  |  |
| Orth Prev. (items) | .0028 | .0525 | .5 | .24 | .9 |  |  |  |  |  | |  |  |
| Intercept (subj) | .0124 | .1116 |  | |  | .0132 | .1150 |  |  | .0157 | | .1254 |  |
| Deg (subj) |  |  |  | |  |  |  |  |  | .0004 | | .0198 | .27 |
| Residual | .0741 | .2723 |  | |  | .0739 | .2718 |  |  | .1043 | | .3230 |  |

*Note*: Invalid Prev.: Invalid preview effect (letter mask vs valid preview). Orth Prev: orthographic preview effect (orthographic vs letter mask preview). Deg: preview degradation. FFD: first fixation duration. SFD: single fixation duration. GD: gaze duration. subj: subjects. Statistically significant *t*-values are formatted in bold.

**Target Word Analyses with Additional Measures (Post-hoc)**

We also did post-hoc analyses on the target word using three additional measures: first-pass skipping probability (the probability of skipping the target word during first-pass reading), regression-in probability (the probability of regressing back to the target word), and regression-out probability (the probability of initiating a regression out of the target word during first-pass reading). The descriptive statistics are presented in Table S8 and GLMM results are shown in Tables S9-S11.

**First-pass skipping probability.** In Experiment 1a, where all words were degraded prior to their fixation, there was a main effect of orthographic preview. This was due to greater skipping probability in the orthographic compared to the letter mask preview condition. Additionally, there was an interaction between the invalid preview effect and degradation. This was due to a reduction in skipping probability in the invalid letter mask condition, but only when the preview was non-degraded. In other words, readers were less likely to skip a letter mask preview when it was not degraded.

Experiment 2a (replication of 1a) also found the same main effect of orthographic preview- orthographic previews were more likely to be skipped than letter mask previews. However, the interaction between the invalid preview effect and degradation did not reach significance. Nevertheless, there was a main effect of invalid preview, which was due to the fact that letter masks were less likely to be skipped than valid previews. Therefore, unlike Experiment 1a, the reduction of skipping probability with letter masks occurred in both the degraded and non-degraded conditions.

Table S8

*First-pass Skipping, Regression-in, and Regression-out Probability on the Target Word in Experiments 1-2 (Standard Deviations in Parenthesis)*

| Degradation | Preview type | p(1^st^ pass Skipping) | p(Regression-in) | p(Regression-out) |
| --- | --- | --- | --- | --- |
| **Experiment 1a** | | | | |
| 0% | valid | .11 (.31) | .20 (.40) | .16 (.37) |
| 0% | phonological | .09 (.29) | .22 (.41) | .15 (.36) |
| 0% | orthographic | .12 (.33) | .22 (.41) | .16 (.37) |
| 0% | letter mask | .07 (.26) | .18 (.38) | .22 (.41) |
| 20% | valid | .10 (.30) | .24 (.43) | .12 (.33) |
| 20% | phonological | .11 (.31) | .23 (.42) | .10 (.30) |
| 20% | orthographic | .11 (.32) | .27 (.45) | .11 (.31) |
| 20% | letter mask | .11 (.32) | .28 (.45) | .13 (.34) |
| **Experiment 1b** | | | | |
| 0% | valid | .14 (.35) | .21 (.41) | .09 (.29) |
| 0% | phonological | .14 (.34) | .20 (.40) | .13 (.34) |
| 0% | orthographic | .12 (.33) | .25 (.43) | .12 (.33) |
| 0% | letter mask | .10 (.30) | .21 (.41) | .20 (.40) |
| 20% | valid | .08 (.27) | .22 (.42) | .18 (.39) |
| 20% | phonological | .08 (.27) | .22 (.41) | .16 (.36) |
| 20% | orthographic | .09 (.29) | .20 (.40) | .18 (.38) |
| 20% | letter mask | .09 (.29) | .23 (.42) | .23 (.42) |
| **Experiment 2a (replication of 1a)** | | | | |
| 0% | valid | .25 (.43) | .20 (.40) | .14 (.35) |
| 0% | orthographic | .19 (.39) | .22 (.42) | .16 (.37) |
| 0% | letter mask | .14 (.35) | .22 (.42) | .24 (.43) |
| 20% | valid | .24 (.43) | .26 (.44) | .12 (.32) |
| 20% | orthographic | .19 (.40) | .25 (.43) | .14 (.34) |
| 20% | letter mask | .17 (.38) | .26 (.44) | .14 (.35) |
| **Experiment 2b (replication of 1b)** | | | | |
| 0% | valid | .24 (.43) | .20 (.40) | .16 (.36) |
| 0% | orthographic | .22 (.42) | .24 (.43) | .17 (.38) |
| 0% | letter mask | .15 (.36) | .25 (.43) | .28 (.45) |
| 20% | valid | .16 (.37) | .20 (.40) | .18 (.39) |
| 20% | orthographic | .15 (.35) | .20 (.40) | .20 (.40) |
| 20% | letter mask | .15 (.36) | .20 (.40) | .21 (.41) |

Table S9

*GLMM Results of First-pass Skipping Probability on the Target Word in Experiments 1-2*

| Fixed effects | Experiment 1a | | |  | Experiment 1b | | | | |  |
| --- | --- | --- | --- | --- | --- | --- | --- | --- | --- | --- |
|  | b | SE | z |  | b | SE | | z | |  |
| Intercept | -2.56 | 0.15 | **-16.91** |  | -2.72 | .17 | | | **-15.72** | |
| Invalid Prev. | -0.14 | 0.16 | -0.89 |  | -.16 | .16 | | | -.98 | |
| Orth Prev. | -0.32 | 0.16 | **-2.03** |  | -.15 | .16 | | | -.91 | |
| Phon Prev. | 0.2 | 0.15 | 1.29 |  | .01 | .16 | | | .09 | |
| Deg | -0.08 | 0.06 | -1.42 |  | .32 | .08 | | | **3.8** | |
| Invalid Prev. x Deg | -0.35 | 0.16 | **-2.18** |  | -.29 | .16 | | | -1.82 | |
| Orth Prev. x Deg | -0.3 | 0.16 | -1.93 |  | -.12 | .16 | | | -.75 | |
| Phon Prev. x Deg | 0.13 | 0.15 | 0.83 |  | -.15 | .16 | | | -.96 | |
| Random effects | Var. | SD | Corr. |  | Var. | | SD | Corr. | |  |
| Intercept (items) | .1881 | .4338 |  |  | .0860 | | .2933 |  | |  |
| Intercept (subj) | .9457 | .9725 |  |  | 1.331 | | 1.154 |  | |  |
| Deg (subj) |  |  |  |  | .0380 | | .1948 | -.64 | |  |
|  |  | |  |  |  | | |  | |  |
| Fixed effects | Experiment 2a | | |  | Experiment 2b | | | | |  |
|  | b | SE | z |  | b | | SE | z | |  |
| Intercept | -1.74 | .13 | **-13.1** |  | -1.99 | | .16 | **-12.26** | |  |
| Invalid Prev. | -.68 | .09 | **-7.88** |  | -.37 | | .09 | **-3.99** | |  |
| Orth Prev. | -.3 | .09 | **-3.38** |  | -.24 | | .09 | **-2.55** | |  |
| Deg | .01 | .05 | .15 |  | .21 | | .04 | **4.95** | |  |
| Invalid Prev. x Deg | -.15 | .09 | -1.73 |  | -.26 | | .09 | **-2.83** | |  |
| Orth Prev. x Deg | -.13 | .09 | -1.5 |  | -.26 | | .09 | **-2.73** | |  |
| Random effects | Var. | SD | Corr. |  | Var. | | SD | Corr. | |  |
| Intercept (items) | .4715 | .6867 |  |  | .3845 | | .6201 |  | |  |
| Deg (items) |  |  |  |  | .0218 | | .1475 | -.20 | |  |
| Intercept (subj) | .7508 | .8665 |  |  | 1.263 | | 1.124 |  | |  |
| Deg (subj) | .0468 | .2164 | -.68 |  |  | |  |  | |  |

*Note*: Invalid Prev.: Invalid preview effect (letter mask vs valid preview). Orth Prev: orthographic preview effect (orthographic vs letter mask preview). Phon Prev.: phonological preview effect (phonological vs. orthographic preview). Deg: preview degradation. subj: subjects. Statistically significant *z*-values are formatted in bold.

In Experiment 1b, where only the target word and the remaining sentence were degraded, there was only a main effect of degradation. This was due to a smaller skipping probability in the degraded compared to the non-degraded condition. In other words, readers were less likely to skip the target when it was degraded.

In Experiment 2b (replication of 1b), there was the same main effect of degradation where degraded previews were less likely to be skipped than non-degraded ones. Additionally, letter mask previews were less likely to be skipped than valid previews (main effect of invalid preview) and orthographic previews were more likely to be skipped than letter mask previews (main effect of orthographic preview). Furthermore, the interaction between the invalid preview effect and degradation was also significant. This occurred because the difference in skipping between the valid and letter mask preview was larger in the non-degraded compared to the degraded condition. Similarly, the interaction between the orthographic preview effect and degradation was also significant, which again occurred because the difference between orthographic and letter mask preview was greater in the non-degraded compared to the degraded condition.

To summarise, target words were less likely to be skipped when they were degraded compared to when they were not degraded. However, this occurred only in the original incremental boundary paradigm (Marx et al., 2015) where just the target and the remaining sentence were degraded (Experiments 1b and 2b). Because the target degradation in this manipulation is perceptually distinct, readers may be less likely to initiate a skip decision. Additionally, there was evidence that readers are less likely to skip letter mask previews, particularly in the non-degraded condition. This may be due to at least two factors: 1) letter masks prevent parafoveal processing of the target, which may make skipping decisions less likely; or 2) if readers notice unusual letter combinations in the parafovea, this may also reduce their likelihood of making a skip. Degrading the masks seemed to reduce (or in some cases eliminate) the effect, which would be consistent with both explanations. Further research in needed to better understand how degradation affects the skipping of different types of degraded previews.

**Regression-in probability.** In Experiment 1a, where all words were degraded prior to their fixation, there was only a main effect of degradation. This was due to a greater probability of regressing to the target word in the degraded compared to the non-degraded condition. Experiment 2a (replication of Experiment 1a) showed identical results- degraded targets were regressed to more often than non-degraded ones, but there were no other significant effects.

Experiment 1b, where only the target word and the remaining sentence were degraded, showed no significant effects. Thus, the experimental manipulation did not influence regressions back to the target. In Experiment 2b (replication of 1b), there was a significant main effect of degradation. However, in contrast to Experiments 1a and 2a, the pattern was reversed and degraded preview paradoxically resulted in *fewer* regressions to the target. The interaction between invalid preview effect and degradation also reached significance. This occurred because the invalid letter mask was regressed to more often than the valid preview, but only when the target word was not degraded. In other words, in the absence of degradation, letter masks led to an increase in regressions back to the target.

To summarise, when all words in the sentence were degraded, parafoveal degradation led to an increase in regressions back to the target. However, when only the target word and remaining sentence were degraded, the results were somewhat inconclusive- one experiment found no evidence for an increase in regressions back to the target, while the second experiment found the opposite result (i.e., degradation causing fewer regression). At present, we remain cautious about interpreting these results until more evidence becomes available.

Table S10

*GLMM Results for Regression-in Probability on the Target Word in Experiments 1-2*

| Fixed effects | Experiment 1a | | |  | Experiment 1b | | | | |  |
| --- | --- | --- | --- | --- | --- | --- | --- | --- | --- | --- |
|  | b | SE | z |  | b | SE | | z | |  |
| Intercept | -1.38 | .10 | **-13.36** |  | -1.47 | .11 | | | **-13.51** | |
| Invalid Prev. | .03 | .12 | .23 |  | .03 | .12 | | | .23 | |
| Orth Prev. | -.1 | .12 | -.84 |  | -.03 | .12 | | | -.27 | |
| Phon Prev. | .13 | .12 | 1.08 |  | .10 | .12 | | | .79 | |
| Deg | -.16 | .04 | **-3.89** |  | < .01 | .04 | | | < .01 | |
| Invalid Prev. x Deg | -.22 | .12 | -1.82 |  | < .01 | .12 | | | .03 | |
| Orth Prev. x Deg | -.16 | .12 | -1.38 |  | -.21 | .12 | | | -1.72 | |
| Phon Prev. x Deg | -.16 | .12 | -1.34 |  | .22 | .12 | | | 1.85 | |
| Random effects | Var. | SD | Corr. |  | Var. | | SD | Corr. | |  |
| Intercept (items) | .3910 | .6253 |  |  | .3004 | | .5480 |  | |  |
| Intercept (subj) | .2329 | .4826 |  |  | .3759 | | .6131 |  | |  |
|  |  | |  |  |  | | |  | |  |
| Fixed effects | Experiment 2a | | |  | Experiment 2b | | | | |  |
|  | b | SE | z |  | b | | SE | z | |  |
| Intercept | -1.35 | .10 | **-13.36** |  | -1.45 | | .09 | **-15.46** | |  |
| Invalid Prev. | .07 | .08 | .85 |  | .14 | | .09 | 1.7 | |  |
| Orth Prev. | .02 | .08 | .25 |  | .04 | | .08 | .45 | |  |
| Deg | -.12 | .04 | **-3.06** |  | .09 | | .04 | **2.15** | |  |
| Invalid Prev. x Deg | .09 | .08 | 1.05 |  | .17 | | .09 | **1.99** | |  |
| Orth Prev. x Deg | -.05 | .08 | -.62 |  | .03 | | .08 | .38 | |  |
| Random effects | Var. | SD | Corr. |  | Var. | | SD | Corr. | |  |
| Intercept (items) | .3262 | .5712 |  |  | .2122 | | .4606 |  | |  |
| Deg (items) |  |  |  |  | .0211 | | .1451 | -.37 | |  |
| Intercept (subj) | .3829 | .6187 |  |  | .3395 | | .5826 |  | |  |
| Deg (subj) | .0218 | .1477 | -.42 |  | .0148 | | .1215 | -.45 | |  |

*Note*: Invalid Prev.: Invalid preview effect (letter mask vs valid preview). Orth Prev: orthographic preview effect (orthographic vs letter mask preview). Phon Prev.: phonological preview effect (phonological vs. orthographic preview). Deg: preview degradation. subj: subjects. Statistically significant *z*-values are formatted in bold.

**Regression-out probability.** In Experiment 1a, where all words were degraded prior to their fixation, there was a main effect of invalid preview. This was due to a greater probability of regressing out of the target word after letter mask previews compared to valid previews. Additionally, the main effect of orthographic preview was also significant. This was due to a greater regression-out probability following letter mask previews compared to orthographic previews. Therefore, regression-out probability after letter masks was higher compared to both valid and orthographic previews. Furthermore, the main effect of degradation was also significant. This was due to a smaller regression-out probability following degraded compared to non-degraded target words.

In Experiment 2a (replication of 1a), all effects were significant. Therefore, Experiment 2a replicated the same effects from Experiment 1a above. In addition to them, the interaction between the invalid preview effect and degradation was also significant. This was due to the fact that the difference in regression-out probability between letter masks and valid previews was greater in the non-degraded compared to the degraded condition. Likewise, the interaction between the orthographic preview effect and degradation was also significant. This again occurred because the difference in regression-out probability between letter masks and orthographic previews was greater in the non-degraded compared to the degraded condition. In other words, degradation seemed to reduce or nearly eliminate the increase in regression-out probability associated with letter masks.

In Experiment 1b, where only the target word and remaining sentence were degraded, there were main effects of invalid preview and orthographic preview. Similar to Experiments 1a and 2a, these were due to greater regression-out probability following letter mask previews compared to both valid and orthographic previews. Additionally, the main effect of degradation was also significant. This was due to *greater* regression-out probability in the degraded compared to the non-degraded condition. Thus, unlike Experiments 1a and 2a, degradation had the opposite effect and led to more regressions out of the target word. Finally, there was also a significant interaction between the invalid preview effect and degradation. Similar to Experiments 1a and 2a, this occurred because the difference in regression-out probability between letter masks and valid previews was greater in the non-degraded compared to the degraded condition.

Experiment 2b (replication of 1b) also replicated the same invalid preview and orthographic preview effects found in the other three experiments. However, the main effect of degradation was not significant. Additionally, the interactions between invalid preview effect and degradation, and orthographic preview effect and degradation were also significant. Similar to the previous experiments, these were again due to the difference between letter mask and valid previews, and letter masks and orthographic previews, being larger in the non-degraded compared to the degraded condition.

To summarise, across the four experiments, participants were more like to regress out of the target word following letter masks previews compared to both valid and orthographic previews. This effect was strongest in the non-degraded condition. Indeed, parafoveal degradation generally seemed to reduce the increase in regression-out probability for letter masks. This could occur if certain properties of the letter mask (e.g., their irregularity) give rise to the increase in regression-out probability, perhaps because readers noticed something unusual in the parafovea. Since degrading the mask also reduces its intelligibility, this may lead to a reduction in regression-out probability. However, at present, this remains just a speculation. Finally, degraded previews also led to a reduction in regression-out probability when all words were degraded in the sentence (Experiments 1a and 2a). This may occur because degradation reduces the visual input quality of the text, which may prompt participants to adopt a more cautious reading strategy where they avoid initiating a regression before the whole text has been “revealed” (i.e., first-pass processing of the sentence is completed and all degradation has disappeared).

Table S11

*GLMM Results for Regression-out Probability on the Target Word in Experiments 1-2*

| Fixed effects | Experiment 1a | | |  | Experiment 1b | | | | |  |
| --- | --- | --- | --- | --- | --- | --- | --- | --- | --- | --- |
|  | b | SE | z |  | b | SE | | z | |  |
| Intercept | -2 | .11 | **-17.57** |  | -1.96 | .14 | | | **-13.75** | |
| Invalid Prev. | .29 | .14 | **2.07** |  | .70 | .14 | | | **4.94** | |
| Orth Prev. | .35 | .14 | **2.46** |  | .52 | .14 | | | **3.83** | |
| Phon Prev. | .10 | .15 | .64 |  | .04 | .15 | | | .27 | |
| Deg | .23 | .06 | **4.15** |  | -.21 | .05 | | | **-4.22** | |
| Invalid Prev. x Deg | .17 | .14 | 1.21 |  | .32 | .14 | | | **2.27** | |
| Orth Prev. x Deg | .08 | .14 | .57 |  | .13 | .14 | | | .99 | |
| Phon Prev. x Deg | < .01 | .15 | .01 |  | -.12 | .15 | | | -.81 | |
| Random effects | Var. | SD | Corr. |  | Var. | | SD | Corr. | |  |
| Intercept (items) | .3209 | .5664 |  |  | .3496 | | .5912 |  | |  |
| Intercept (subj) | .3299 | .5744 |  |  | .7718 | | .8785 |  | |  |
| Deg (subj) | .0062 | .0789 | .56 |  |  | |  |  | |  |
|  |  | |  |  |  | | |  | |  |
| Fixed effects | Experiment 2a | | |  | Experiment 2b | | | | |  |
|  | b | SE | z |  | B | | SE | z | |  |
| Intercept | -1.89 | .08 | **-22.92** |  | -1.66 | | .11 | **-15.4** | |  |
| Invalid Prev. | .48 | .10 | **5.06** |  | .50 | | .09 | **5.6** | |  |
| Orth Prev. | .27 | .09 | **2.94** |  | .40 | | .09 | **4.56** | |  |
| Deg | .21 | .05 | **4.45** |  | .05 | | .05 | 1 | |  |
| Invalid Prev. x Deg | .25 | .10 | **2.65** |  | .32 | | .09 | **3.6** | |  |
| Orth Prev. x Deg | .23 | .09 | **2.58** |  | .32 | | .09 | **3.62** | |  |
| Random effects | Var. | SD | Corr. |  | Var. | | SD | Corr. | |  |
| Intercept (items) | .3088 | .5557 |  |  | .3993 | | .6319 |  | |  |
| Deg (items) | .0502 | .2241 | -.03 |  | .0177 | | .1331 | -.17 | |  |
| Intercept (subj) | .1516 | .3894 |  |  | .4029 | | .6347 |  | |  |
| Deg (subj) |  |  |  |  | .0208 | | .1443 | -.70 | |  |

*Note*: Invalid Prev.: Invalid preview effect (letter mask vs valid preview). Orth Prev: orthographic preview effect (orthographic vs letter mask preview). Phon Prev.: phonological preview effect (phonological vs. orthographic preview). Deg: preview degradation. subj: subjects. Statistically significant *z*-values are formatted in bold.

**Breakdown by Degradation Display Change Awareness in Experiment 1b**

In Experiment 1b, 85.9 % of all participants reported noticing degraded display changes, while the remaining 14.1 % did not report noticing them. Therefore, one could argue that those participants who did not notice the degraded display changes may exhibit results that mimic the pattern of results found in Experiment 1a- namely, that degradation would not add any costs of its own and that it will reduce the preview costs from the invalid letter mask condition. The breakdown of results for participants who noticed and participants who did not notice the degraded changes is presented in Figure S1. The target word analysis from the main paper was repeated by adding participant display change awareness as a factor to the model (contrast coding: -1 = noticed degradation; 1= did not notice degradation). However, neither display change awareness nor any of its interactions with the remaining two factors (target word preview and degradation) reached statistical significance, all |*t*|s ≤ 1.85. Therefore, degradation awareness did not modulate the results from Experiment 1b. The same analysis was not done for Experiment 2b (replication of 1b) since even fewer participants (6.6%) reported no awareness of degraded display changes.

The results above may not necessarily be surprising since the group with participants that did not notice degraded display changes was much smaller than the one that did notice them, and the mean estimates were naturally much noisier. Additionally, this analysis did not consider differences in awareness at the trial level. Therefore, future research with the display-change detection paradigm (Angele et al., 2016; Slattery, Angele, & Rayner, 2011) might be better suited to answer this question. However, this would still not remove the bias of having two groups of very unequal sample sizes.


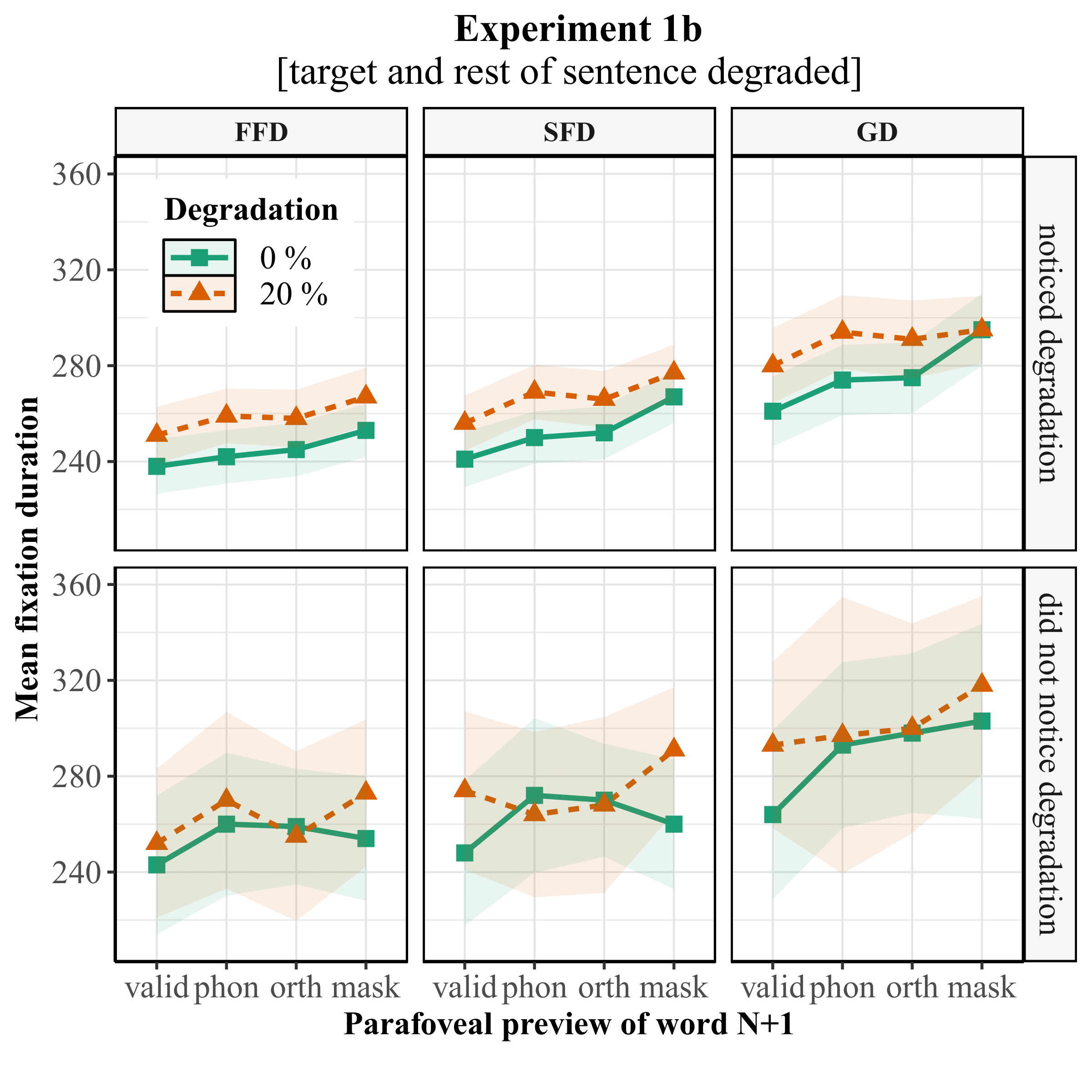


*Figure S1*. Breakdown of the target word results in Experiment 1b for participants who noticed and participants who did not notice degraded display changes. valid: valid preview; phon: phonological preview; orth: orthographic preview; mask: letter mask preview. FFD: first fixation duration. SFD: single fixation duration; GD: gaze duration. Shading indicates ± 1 SE.

**Reading Stimuli from Experiment 1**

Reading stimuli used in Experiments 1a-1b. The target word and the preview condition are formatted in bold. The four preview conditions are in the following order: **valid preview** (the target word itself) / **phonological preview**/ **orthographic preview**/ **letter mask**.

| 1. The reporter announced that no people were **hurt/ hirt/ hort/ iomk** in the car accident this morning. |
| --- |
| 1. The client asked for another **type/ tipe/ tepe/ djya** of dress that is more suitable for a wedding. |
| 1. Jesse had to remove **dirt/ dert/ dort/ tkuh** from his clothes after he cycled home in the rain. |
| 1. John surprised his wife with a designer **purse/** **perse/ porse/ yezou** that he bought on his trip to Italy. |
| 1. In the past year, the local police took **stern/ sturn/ storn/ efoma** measures to improve safety on the roads. |
| 1. The painting depicted how the two streams **merge/ murge/ morge/ novyu** to form the magnificent river. |
| 1. The government tried to support the recent **surge/ sirge/ sorge/ romja** of interest in solar power technology. |
| 1. The money for a new library was raised by the local **church/ chirch/ charch/ edavab** and members of the public. |
| 1. The speaker tried to make a joke after he let out a loud **burp/ birp/ borp/ havg** during his presentation. |
| 1. In their lesson, the students were taught what a proper **noun/ nown/ noin/ zusm** was and how to spell it. |
| 1. Jessica bought a fancy **skirt/ skurt/ skart/ utlul** that she wanted to wear to the beach party. |
| 1. By joining the army, the young man hoped he could **serve/ surve/ sorve/ zasoa** and protect his country. |
| 1. Not long after their **worst/ wirst/ warst/ sawal** rain storm in the mountain, the campers decided to go home. |
| 1. For Sam, getting a well-paid job helped **purge/ perge/ parge/ gomyo** the guilt of buying an expensive car. |
| 1. After the summer **term/ tirm/ tarm/ fuwa** ended, the students went on a holiday in Spain. |
| 1. Katy put on her new dress and did a quick **twirl/ twurl/ tworl/ iuloi** to show it to her mother. |
| 1. The teen made a dramatic **plea/ plee/ pler/ qdam** to the judge in hopes of avoiding a severe sentence. |
| 1. The tailor repaired the ripped **sleeve/ sleave/ slerve/ eiusro** of the expensive leather jacket. |
| 1. The couple ordered a chimney **sweep/ sweap/ swerp/ euocg** before they moved into their new house. |
| 1. While on the road, the car made a strange **jerk/ jurk/ jark/ yasb** before the engine died. |
| 1. Despite the security, the youngsters managed to quietly **sneak/ sneek/ snerk/ auavd** into the movie theatre. |
| 1. Michael made a mental note to bring the broken **steam/ steem/ sterm/ okanw** hoover to the repair shop. |
| 1. The restaurant offered tender **beef/ beaf/ berf/ tuvl** steak as a meal of the day. |
| 1. The astronomer investigated the strange **burst/ birst**/ **barst/ fecoh** of energy that was detected from Earth. |
| 1. Because the weather seemed **bleak/ bleek/ blerk/ kiaml** and uninviting, the old man decided to stay at home. |
| 1. Thanks to her long **sleek/ sleak/ slerk/ etozb** hair, the actress was often invited to be in commercials. |
| 1. Because the road looked **steep/ steap/ sterp/ okonj** and difficult to climb, the cyclists pushed their bikes. |
| 1. Jane liked the crisp **veal/ veel/ verl/ nusf** pie that her grandmother prepared for the holidays. |
| 1. The skillful camper was able to quickly **cleave/ cleeve/ clerve/ okazsa** the logs with his hatchet. |
| 1. Unfortunately, the large **bleach/ bleech/ blerch/ dfomzt** stains had completely ruined the new clothes. |
| 1. The cat chased the brown **shrew/ shrue/ shree/ akeuv** that had strayed from the forest looking for food. |
| 1. The man spoke with a distinct **slur/ slir/ slar/ ukaz** which made the police suspicious that he was drunk. |
| 1. Sarah always found it embarrassing to see people **flirt/ flert/ flort/ ldfeh** on the street. |
| 1. The children told their story in mutual **mirth/ merth/ morth/ ufalk** while sitting next to the camp fire. |
| 1. The lorry driver made a sudden **swerve/ swurve/ sworve/ ueuneo** as he was about to miss the motorway exit. |
| 1. The shaky boat made Carla's head **swirl/ swurl/ sworl/ eebot** as she struggled to keep her balance. |
| 1. The boy had a tiny **smirk/ smurk/ smark/ euboi** on his face after he saw his mother scold his brother. |
| 1. The admiral would never **speak/ speek/ sperk/ oqosb** badly of his superiors even if he didn't agree with them. |
| 1. Before her morning run, Ana put on a thick **fleece/ fleese/ fleeme/ bhuzzu** jacket to keep herself warm. |
| 1. The young athlete was happy after he finished **third/ thurd/ thard/ hdkah** in the international competition. |
| 1. The workers gave the sofa a good **heave/ heeve/ herve/ iuvwu** and moved it up the staircase. |
| 1. The fact that the next competition was on their home **turf/ terf/ tarf/ hozi** raised the swimmers' morale. |
| 1. The new type of toothpaste promised white **teeth/ teath/ terth/ hasfk** just after a few days of use. |
| 1. The instructor demonstrated how to properly **hurl/ hirl/ harl/ kewf** a cricket ball to the children. |
| 1. The little girl danced with utter **glee/ glea/ gler/ qtom** to the sounds of pop music. |
| 1. After Nick finished the race first, there was a small **gleam/ gleem/ glerm/ jkawn** of triumph in his eyes. |
| 1. The man decided to wear a blue **tweed/ twead/ twerd/ kuamk** jacket for his job interview. |
| 1. The laws in the 20th century meant that people could no longer **lynch/ linch/ lanch/ bpook s**uspects of crime. |
| 1. The surgeon was careful to avoid **nerve/** **nurve/ narve/ cuzoa** tissue while performing the operation. |
| 1. The possibility of eternal **youth/ youth/ yoath/ guwkt** has fascinated mankind for centuries. |
| 1. According to the announcement, the expected **sleet/ sleat/ slert/ ubork** may cause disturbances on the road. |
| 1. The climber used a short **coil/ koil/ hoil/ wabk** of rope to secure his tent in the mountain. |
| 1. The recently hired **dean/ deen/ dern/ tarw** made a commitment to improve the diversity in the department. |
| 1. The students were expected to somehow **glean/ gleen/ glern/ jionv** the information for their class report. |
| 1. The client was advised to contact **tech/ teck/ tece/ domk** support if the software is not working properly. |
| 1. Rob's teachers were impressed by his constant **thirst/ thurst/ thorst/ ldbaeb** for knowledge. |
| 1. The landlady asked the tenants to keep the house **clean/ cleen/ clern/ edasz** and tidy after they move in. |
| 1. The old lady thanked the friendly **nurse/ nerse/ narse/ zawaa** who was checking on her every other week. |
| 1. Dan challenged his bigger brother to finish the game without **cheat/ cheet/ chert/ adomh** codes of any kind. |
| 1. Karen got a small scar on her right **cheek/ cheak/ cherk/ otami** after she fell off her bike. |
| 1. The sailor secured his boat to the brass **cleat/ cleet/ clert/ akusb** on the dock and then headed home. |
| 1. The CEO gave his secretary several **terse/ turse/ tarse/ baceu** instructions and then left the office. |
| 1. The cottage was surrounded by dense **fern/ furn/ farn/ kaza** vegetation that was not trimmed in years. |
| 1. Jasmine found a great **deal/ deel/ derl/ famb** online and treated herself to a spa holiday. |
| 1. The artist wore a satin dress with a slight **sheen/ shean/ shern/ adozc** that was made for the performance. |
| 1. The children's most vivid memory of the zoo was the wild **lynx/ linx/ lonx/ bgae** sitting on a tree. |
| 1. The report warned that without protection the species would **cease/ ceese/ cerse/ rovwu** to exist in a decade. |
| 1. Marina had a good **weep/ weap/ werp/ nacg** after watching the sad movie drama last night. |
| 1. Brad was a very **meek/ meak/ meck/ vowf** person who never got into arguments with his colleagues. |
| 1. Mary's children were old enough to earn their **keep/ keap/ kerp/ iamy** and decided to rent their own flat. |
| 1. The girl always tried to keep her clothes **neat/ neet/ nert/ zawb** and clean when playing outside. |
| 1. The news of the rising **gross/ grose/ grosh/ paune** sales inspired confidence in the future of the company. |
| 1. Lisa spent her holiday in a social **whirl/ whurl/ wharl/ akdei** of activities that freed her mind from work. |
| 1. Betty thought that the light **teal/ teel/ terl/ lozt** curtains would be a good addition to her bedroom. |
| 1. According to some people, investing in real estate is well **worth/ wirth/ warth/ cusbt** the money. |
| 1. The children were afraid when they saw the ocean **seethe/ seathe/ serthe/ cosifu** restlessly beneath them. |
| 1. After he left **work/ work/ wark/ muzb** on Friday, Alex headed to the pub to catch up with his friends. |
| 1. The new movie features a peaceful **dwarf/ dworf/ dwarf/ kauwb** who tries to unite his kingdom. |
| 1. The scientists could finally **prove/ pruve/ prive/ yeemo** that the newly discovered substance is not toxic. |
| 1. Visiting the dentist always made Zoe's stomach **churn/ chirn/ charn/ ebano** for the whole morning. |

**Reading Stimuli from Experiment 2**

Additional reading stimuli used in Experiments 2a-2b. The complete corpus included all sentences used in Experiment 1 (see above), with the addition of the new sentences below that were written specifically for Experiment 2. This led to a total of 138 sentences. The target word and the preview condition are formatted in bold. The three preview conditions are in the following order: **valid preview** (the target word itself) / **orthographical preview**/ **letter mask** (note that the phonological preview condition was removed from Experiment 2).

| 1. The new gardener forgot to trim the green **bush/ bish/ denl** near the front door of the manor. |
| --- |
| 1. Susan could not believe the record **cold/ colc/ mebh** weather they had been having all week. |
| 1. The students were happy that the first **part**/ **parl**/ **jonb** of the exam had multiple-choice questions. |
| 1. The comedian spoke with a funny **lisp/ losp/ kbeq** during all his stand-up acts. |
| 1. The tourists scrambled to escape the path of the charging **herd**/ **herf**/ **lumh** of water buffalo. |
| 1. The couple got to see zebras running **wild**/ **wilk**/ **ahbf** during their safari holiday. |
| 1. The barber was trying a new scented **talc**/ **tilc**/ **duhu** and his customers liked it. |
| 1. Tommy had placed a large bet on the losing **team**/ **teap**/ **bovn** and didn’t have the money to cover it. |
| 1. Susan slipped on the bottom **step**/ **slep**/ **akoj** of the spiral staircase and twisted her ankle. |
| 1. The newly forged sword's edge was still **blunt**/ **blunk**/ **ibacf** and needed to be sharpened. |
| 1. The very strong **chain/ chaim/ utolu** was made by the most expert blacksmith. |
| 1. The book ended with the sudden **death/ deach/ iockd** of one of the main protagonists. |
| 1. The old woman complained that the cold **draft**/ **drafs**/ **luohb** was making her back ache. |
| 1. George had only come **fifth/ fifth/ tldbb** in the horse race but was still happy with his result. |
| 1. Harriet was frightened by the evil **ghost/ ghist/ pbazk** she thought lived in her house. |
| 1. Karl knew he couldn't **trust/ trost/ boawk** anyone with his secret. |
| 1. Joe was upset that he had lost his special **spoon**/ **spoot**/ **eguzv** which had been gifted to him. |
| 1. Dolly the sheep was the world's first **clone/ clome/ udavu** of a mammal to survive infancy. |
| 1. The TV chef always made sure he sharpened his carving **knife/ knike/ dohtu** before the show. |
| 1. The inspector noticed a small **crack/ creck/ eouwh** in the building's foundation that needed repairing. |
| 1. The police found an empty **flask/ flosk/ hiomb** beneath the front seat that smelled of liquor. |
| 1. The married couple decided to go on a luxury **cruise/ cruive/ saodoa** for their second honeymoon. |
| 1. Johan gave his younger brother a fist **bump/ bemp/ dosj** after he won the swimming competition. |
| 1. The team needs to play much better during the last **nine/ nipe/ ofoa** minutes if they want to win. |
| 1. The sound from the large **horn/ harn/ kuca** echoed through the green valley. |
| 1. The beauty queen wore a gorgeous **sash/ sush/ zuwk** over her elegant red gown. |
| 1. The teacher told the child to first **spit**/ **spim/ aqld** out his gum and then answer the question. |
| 1. He bought a large plastic **rake/ ruke/ voiu** to gather the fallen leaves in his front garden. |
| 1. The athlete believed she would **reap/ reat/ zovy** the rewards of all her training when she competed. |
| 1. The child enjoyed eating the ripe **plum/ prum/ yfos** she had picked from the tree. |
| 1. The CEO arrived just before **noon/ noom/ serz** and took the seat at the head of the table. |
| 1. The man applied **musk/ musp/ ravh** to the collar of his shirt before his date. |
| 1. The engineers made sure the dynamite would **blast/ blask/ kdevi** straight through the rock. |
| 1. James loved a tavern **brawl/ blawl/ feumb** despite having often landed in hospital afterwards. |
| 1. The boy knew he shouldn't have eaten **chalk**/ **chalf**/ **ubufh** again and began to cry. |
| 1. The prospector knew that his land **claim**/ **claic**/ **udedo** would require a lot of paperwork. |
| 1. The stranger began to feel **faint/ failt/ leduk** at the sight of his own blood. |
| 1. Jenny saw the first snow **flake**/ **flate**/ **bhodu** of winter and braced herself for the cold weather. |
| 1. All the towns people knew about the great **flood**/ **flop/ hiezi** that had destroyed the town in 1912. |
| 1. The fraudster knew that he could **frame/ frape/ tuosu** his brother for his many crimes. |
| 1. The deer were grazing in the grassy **glade/ glane/ ptobo** in the middle of the woods. |
| 1. The detective found the horrible **truth/ treth/ boekb** when he opened the door. |
| 1. The sailor fastened his large **trunk/ trenk/ laomh** so that it would not shift when the boat swayed. |
| 1. Carmen thought her husband’s gift was very **sweet/ sweec/ aouzk** and she thanked him for it. |
| 1. The quarry produced **slate/ swate/ okufu** to be used in the construction of houses. |
| 1. The roof had fallen in after a support **strut/ strot/ ekoob** had been damaged by termites. |
| 1. The car raced around the corner with great **speed/ speeb/ ujark** and roared up the road. |
| 1. The accident victim's **pulse**/ **pulve**/ **yatau** was shallow but steady when he was put in the ambulance. |
| 1. The bandit was lying **prone/ pryne/ yauza** so that the police would not see him. |
| 1. It was unwise to go out at night as the salt **marsh/ mersh/ cenub** around the house was dangerous. |
| 1. Abby was going to take down the ugly **shelf/ shelk/ abudk** in the corner of her living room. |
| 1. The restaurant critic noted the clean **plate/ plafe/ jdeku** and silverware on the table. |
| 1. Even though it was just a short **drive/ dries/ hufoa** to the store, Mike ran out of petrol. |
| 1. The bully watched the child **flinch**/ **flince**/ **kdtueb** when he acted like he was going to punch him. |
| 1. When Jane saw the slow **clench/ clonch/ atoxob** of his fists, she realised that the man is getting angry. |
| 1. They lost millions when the wealthy **client/ crient/ akfusd** decided to take his business elsewhere. |
| 1. They saw the judge **slouch**/ **slooch**/ **aierwb** in her chair just before she fell asleep. |
| 1. Edmond thought the music had a really funky **groove/ groose/ quamsu** that made him want to dance. |

References

Angele, B., Slattery, T. J., & Rayner, K. (2016). Two stages of parafoveal processing during reading: Evidence from a display change detection task. *Psychonomic Bulletin & Review*, *23*(4), 1241–1249. https://doi.org/10.3758/s13423-015-0995-0

Brothers, T., Hoversten, L. J., & Traxler, M. J. (2017). Looking back on reading ahead: No evidence for lexical parafoveal-on-foveal effects. *Journal of Memory and Language*, *96*, 9–22. https://doi.org/10.1016/j.jml.2017.04.001

Drieghe, D. (2011). Parafoveal-on-foveal effects on eye movements during reading. In S. P. Liversedge, I. D. Gilchrist, & S. Everling (Eds.), *The oxford handbook of eye movements* (pp. 1–18). Oxford, UK: Oxford University Press. https://doi.org/10.1093/oxfordhb/9780199539789.013.0046

Green, P., & Macleod, C. J. (2016). SIMR: An R package for power analysis of generalized linear mixed models by simulation. *Methods in Ecology and Evolution*, *7*(4), 493–498. https://doi.org/10.1111/2041-210X.12504

Hutzler, F., Schuster, S., Marx, C., & Hawelka, S. (2019). An investigation of parafoveal masks with the incremental boundary paradigm. *PLoS ONE*, *14*(2), 1–26. https://doi.org/10.1371/journal.pone.0203013

Inhoff, A. W., Starr, M., & Shindler, K. L. (2000). Is the processing of words during eye fixations in reading strictly serial? *Perception and Psychophysics*, *62*(7), 1474–1484. https://doi.org/10.3758/BF03212147

Marx, C., Hawelka, S., Schuster, S., & Hutzler, F. (2015). An incremental boundary study on parafoveal preprocessing in children reading aloud: Parafoveal masks overestimate the preview benefit. *Journal of Cognitive Psychology*, *27*(5), 549–561. https://doi.org/10.1080/20445911.2015.1008494

Slattery, T. J., Angele, B., & Rayner, K. (2011). Eye movements and display change detection during reading. *Journal of Experimental Psychology: Human Perception and Performance*, *37*(6), 1924–1938. https://doi.org/10.1037/a0024322

Starr, M. S., & Inhoff, A. W. (2004). Attention allocation to the right and left of a fixated word: Use of orthographic information from multiple words during reading. *European Journal of Cognitive Psychology*, *16*(1–2), 203–225. https://doi.org/10.1080/09541440340000150

Vasilev, M. R., Slattery, T. J., Kirkby, J. A., & Angele, B. (2018). What are the costs of degraded parafoveal previews during silent reading? *Journal of Experimental Psychology: Learning, Memory, and Cognition*, *44*(3), 371–386. https://doi.org/10.1037/xlm0000433

Veldre, A., & Andrews, S. (2018). How does foveal processing difficulty affect parafoveal processing during reading? *Journal of Memory and Language*, *103*(July), 74–90. https://doi.org/10.1016/j.jml.2018.08.001
